# Dual targeting of CDK4/6 and CDK7 augments tumor response and antitumor immunity in breast cancer models

**DOI:** 10.1172/JCI188839

**Published:** 2025-08-12

**Authors:** Sungsoo Kim, Eugene Son, Ha-Ram Park, Minah Kim, Hee Won Yang

**Affiliations:** 1 Department of Pathology and Cell Biology and; 2 Herbert Irving Comprehensive Cancer Center, Columbia University, New York, New York, USA.

**Keywords:** Cell biology, Oncology, Breast cancer

## Abstract

Cyclin-dependent kinase 4/6 inhibitors (CDK4/6i) have transformed the treatment landscape for hormone receptor^+^ (HR^+^) breast cancer. However, their long-term efficacy is limited by acquired resistance, and CDK4/6i monotherapy remains ineffective in triple-negative breast cancer (TNBC). Here, we demonstrate that dual inhibition of CDK4/6 and CDK7 is a promising strategy to overcome therapeutic resistance in both HR^+^ and TNBC models. Kinetic analyses revealed that CDK7 inhibitors (CDK7i) primarily impair RNA polymerase II–mediated transcription rather than directly targeting cell cycle CDKs. This transcriptional suppression attenuated E2F-driven transcriptional amplification, a key mechanism for developing CDK4/6i resistance following the degradation of the retinoblastoma protein. Consequently, combining CDK7i at minimal effective concentrations with CDK4/6i potently inhibited the growth of drug-resistant tumors. Furthermore, dual CDK4/6 and CDK7 inhibition stimulated immune-related signaling and cytokine production in cancer cells, promoting antitumor immune responses within the tumor microenvironment. These findings provide mechanistic insights into CDK inhibition and support the therapeutic potential of combining CDK7i with CDK4/6i for breast cancer treatment.

## Abstract 2

Combined CDK4/6 and CDK7 inhibition suppresses breast tumor growth and drug resistance while enhancing anti-tumor immunity, offering a promising therapeutic strategy.

## Graphical abstract

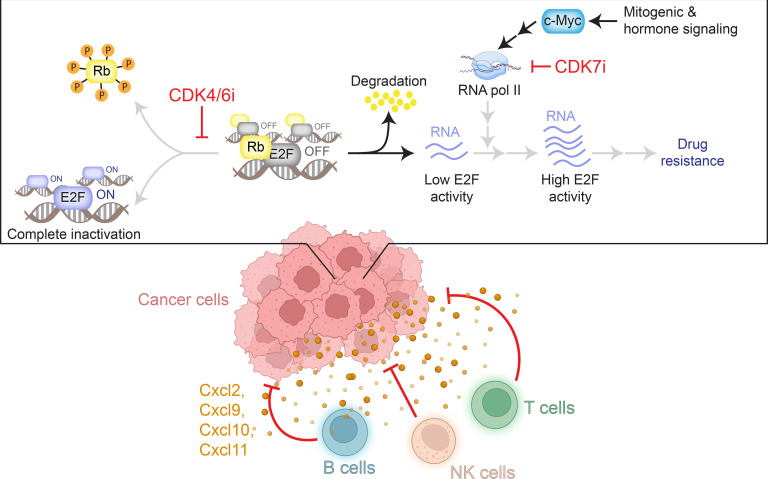

## Introduction

Metastatic breast cancer remains a leading cause of cancer-related mortality among women worldwide (1). A central mechanism driving breast cancer progression involves the hyperactivation of cyclin-dependent kinases 4 and 6 (CDK4/6), which are essential regulators of cell cycle entry (2, 3). This insight has led to major advances in targeted therapy, particularly for hormone receptor^+^ (HR^+^)/human epidermal growth factor receptor 2–negative (HER2^–^) breast cancer, which comprises approximately 70% of all breast cancer cases (3–5). The current standard first-line treatment for HR^+^/HER2^–^ metastatic breast cancer is the combination of CDK4/6 inhibitors (CDK4/6i) and endocrine therapy (ET). However, about 30% of patients develop resistance within 2 years, limiting the long-term therapeutic efficacy (6, 7). Although PI3Kα inhibitors may offer a potential second-line option for patients with *PIK3CA*-mutant tumors (8), a broadly effective treatment strategy for those who progress after CDK4/6i therapy has yet to be established. Additionally, CDK4/6i monotherapy has shown limited efficacy in triple-negative breast cancer (TNBC), an aggressive subtype for which effective targeted therapies remain lacking (9, 10).

In HR^+^/HER2^–^ breast cancer, specific genetic mutations such as *FAT1* loss and *PTEN* loss have been implicated in CDK4/6i resistance (7, 11–15). However, approximately 70% of resistance cases arise without new somatic mutations, highlighting the importance of nongenetic mechanisms (6, 7). The primary substrate of CDK4/6 is the retinoblastoma (Rb) protein, which inhibits cell cycle entry by sequestering E2F transcription factors (4, 5). Accordingly, Rb loss is the most well-established mechanism of CDK4/6i resistance (2–4). While *Rb* loss-of-function mutations are relatively rare in HR^+^/HER2^–^ breast cancer (4.7%) (7, 16), they are more prevalent in TNBC (20%–30%) (17, 18). Nevertheless, approximately 70% of TNBC tumors retain a functional Rb/E2F pathway, indicating that a substantial proportion may respond to CDK4/6i-based therapies.

Our recent studies have identified an alternative mechanism for Rb inactivation through proteasomal degradation triggered by CDK4/6 inhibition in breast cancer (19–21). However, this passive Rb inactivation results in insufficient E2F activity, which needs amplification to drive CDK4/6i resistance. The transcription amplifier c-Myc, frequently overexpressed in TNBC (22), plays a pivotal role in enhancing E2F transcriptional activity. However, targeting c-Myc is challenging due to the lack of a defined drug-binding pocket. Since c-Myc promotes transcription by increasing RNA polymerase II activity (23–26), targeting the transcriptional machinery may provide an alternative strategy to inhibit c-Myc function and CDK4/6i resistance in tumors with an intact Rb/E2F pathway.

CDK7 is uniquely positioned in cancer biology due to its dual role in regulating both mRNA transcription and cell cycle progression through the phosphorylation of RNA polymerase II and cell cycle CDKs, respectively (27, 28). This dual functionality has made CDK7 inhibitors (CDK7i) attractive as standalone therapies in breast cancer, potentially eliminating the need for additional CDK-targeted agents (29). Recent clinical trials have evaluated the maximum tolerated dose of CDK7i as a second-line therapy in HR^+^/HER2^–^ breast cancer patients who progressed on CDK4/6i and ET, as well as a first-line option for TNBC (ClinicalTrials.gov NCT03363893 and NCT04247126) (30). Furthermore, a previous study suggested that combining CDK7i with ET may provide an effective second-line strategy to overcome treatment resistance in HR^+^/HER2^–^ breast cancer (31). Despite these promising advances, the precise effect of CDK7 inhibition in breast cancer remains incompletely understood. Notably, given that CDK activity depends on cyclin expression, maximum inhibition of RNA polymerase II by CDK7i can indirectly influence cell cycle progression, complicating the mechanistic interpretation of its downstream effects.

In this study, we demonstrate that CDK7i primarily suppresses RNA polymerase II–mediated transcription, rather than directly targeting cell cycle CDKs. We propose that dual inhibition of CDK4/6 and CDK7 represents a rational therapeutic strategy, potentially serving as a first-line approach for TNBC and as a second-line option for HR^+^/HER2^–^ breast cancer that has progressed on CDK4/6i and ET. Our findings provide mechanistic insights into CDK inhibition and highlight the therapeutic potential of combining CDK7i and CDK4/6i to overcome drug resistance in breast cancer models.

## Results

### RNA polymerase II as the primary target of CDK7i and the limitations of CDK inhibitor monotherapy in TNBC.

**Figure 1:**
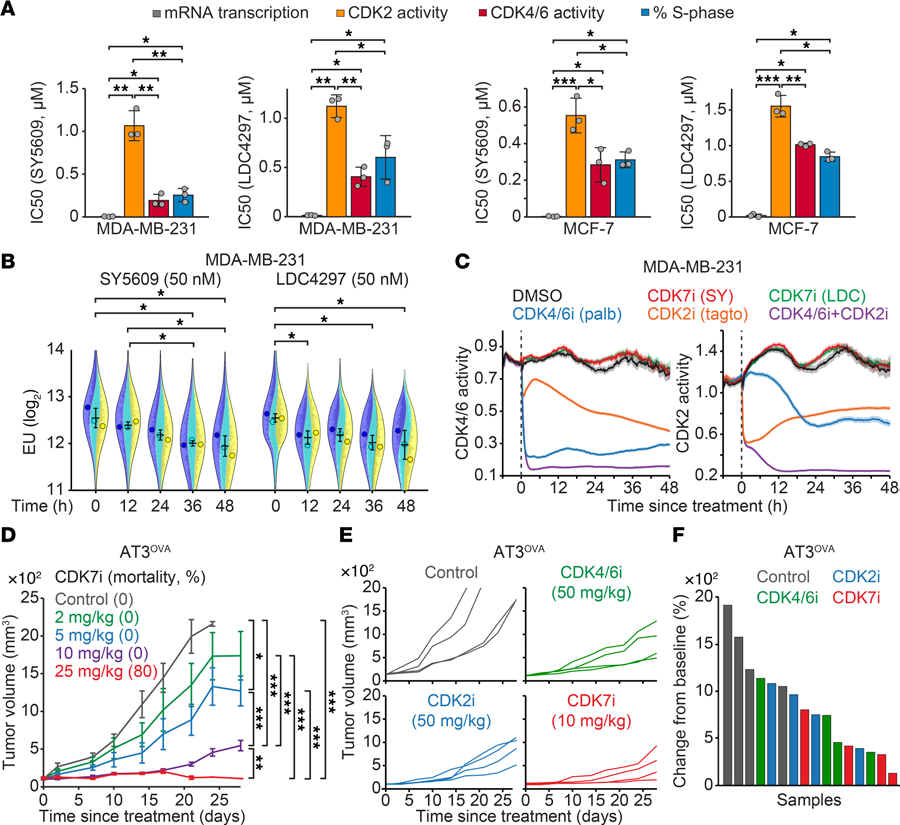
Impact of CDK7i and CDK inhibitor monotherapies on breast cancer. (**A**) IC50 of CDK7i SY5609 and LDC4297 on mRNA transcription rates, CDK2 and CDK4/6 activities, and the percentage of S phase cells in MDA-MB-231 and MCF-7 cells following 48 h treatment. Data are shown as means ± SD (*n* = 3 biological replicates). *P* values were calculated by 1-way ANOVA with post hoc Tukey’s test (*P ≤ 0.05; **P ≤ 0.001; ***P ≤ 0.0001). (**B**) Violin plots of EU incorporation in MDA-MB-231 cells treated with either SY5609 (50 nM) or LDC4297 (50 nM). Cells were randomly selected for 1,000 cells per condition in each replicate. Data are shown as means ± SD (*n* = 3 biological replicates). *P* values were calculated by 2-way ANOVA with post hoc Tukey’s test (*P ≤ 0.05). (**C**) Averaged live-cell traces of CDK4/6 and CDK2 activity in MDA-MB-231 cells treated with DMSO, SY5609 (50 nM), LDC4297 (50 nM), palbociclib (1 μM), tagtociclib (5 μM), or palbociclib + tagtociclib. Data are shown as mean ± 95% CIs (*n* > 1,800 cells/condition). (**D**) Tumor growth curves of AT3^OVA^ syngeneic mouse models treated with vehicle or increasing doses of SY5609 (2, 5, 10, or 25 mg/kg). Data are shown as means ± SEM (*n* = 5 mice/group). *P* values were calculated by a mixed-effect model (*P ≤ 0.05; **P ≤ 0.001; ***P ≤ 0.0001). (**E** and **F**) Individual (**E**) and final (**F**) tumor volumes of AT3^OVA^-bearing C57BL/6J mice treated with vehicle, palbociclib (50 mg/kg), tagtociclib (50 mg/kg), or SY5609 (10 mg/kg).

We investigated the effect of 2 specific CDK7 inhibitors (SY5609 and LDC4297) on mRNA transcription, cell cycle CDK activity, and cell proliferation in TNBC (MDA-MB-231) and HR^+^/HER2^–^ (MCF-7) cell lines. We used live-cell sensors to monitor CDK4/6 and CDK2 activities (32, 33), while mRNA transcription and cell proliferation (% S phase cells) were assessed by 5-ethynyl uridine (EU) and 5-ethynyl-2′-deoxyuridine (EdU) incorporation, respectively. Cells were treated with various concentrations of CDK7i for 48 h to determine their half-maximal inhibitory concentration (IC50). We found that CDK7i exhibited significantly lower IC50 values for mRNA transcription (3–16 nM) compared with those required to inhibit CDK4/6 and CDK2 activities or cell proliferation (152–1,557 nM) (Figure 1A and Supplemental Figure 1; supplemental material available online with this article; https://doi.org/10.1172/JCI188839DS1). Notably, kinetic analyses revealed that CDK7i treatment reduced RNA polymerase II phosphorylation and mRNA transcription without impacting CDK4/6 or CDK2 activity (Figure 1, B and C, and Supplemental Figure 2, A–F). Furthermore, CDK7i treatment did not alter cell cycle phase distribution (Supplemental Figure 2G). Given the reported on-target IC50 values for CDK7i (SY5609, 0.07 nM; LDC4297, 0.13 nM) (34, 35), our data indicate that CDK7i primarily targets mRNA transcription rather than cell cycle CDK activity.

To model the clinical evaluation of maximum tolerated doses of CDK7i in breast cancer (30), we assessed the dose-dependent antitumor efficacy of CDK7i in a syngeneic mouse TNBC model by orthotopically injecting AT3 cells expressing ovalbumin (AT3^OVA^) into the mammary fat pad of C57BL/6J mice (36). Once tumors reached an average volume of 100 mm³, mice were treated with escalating doses of CDK7i (SY5609; 0, 2, 5, 10, and 25 mg/kg). CDK7i treatment induced a dose-dependent antitumor response (Figure 1D and Supplemental Figure 3A). However, the 25 mg/kg dose was associated with toxicity, as evidenced by weight loss and mortality (Supplemental Figure 3B). At 10 mg/kg, tumor growth was delayed, but continued progression was observed during the treatment period, suggesting limited durability of the response at this dose. These results indicate that the therapeutic efficacy of CDK7i monotherapy in TNBC may be constrained by both dose-limiting toxicity and insufficient long-term tumor control. To further explore the potential of CDK inhibitor monotherapies in TNBC, we assessed CDK7i, CDK4/6i, and CDK2i as monotherapies in the AT3^OVA^ model. Like CDK4/6, CDK2 is a crucial cell cycle regulator, particularly in the G1/S transition (37, 38). Although each monotherapy delayed tumor progression with varying efficacy, TNBC tumors developed resistance to all CDK inhibitor treatments (Figure 1, E and F, and Supplemental Figure 3C). These findings indicate the limitations of CDK inhibitor monotherapies in TNBC treatment, underscoring the need for combination therapies to achieve durable antitumor effects.

### Targeting transcriptional amplification with CDK7i prevents CDK4/6i resistance.

**Figure 2:**
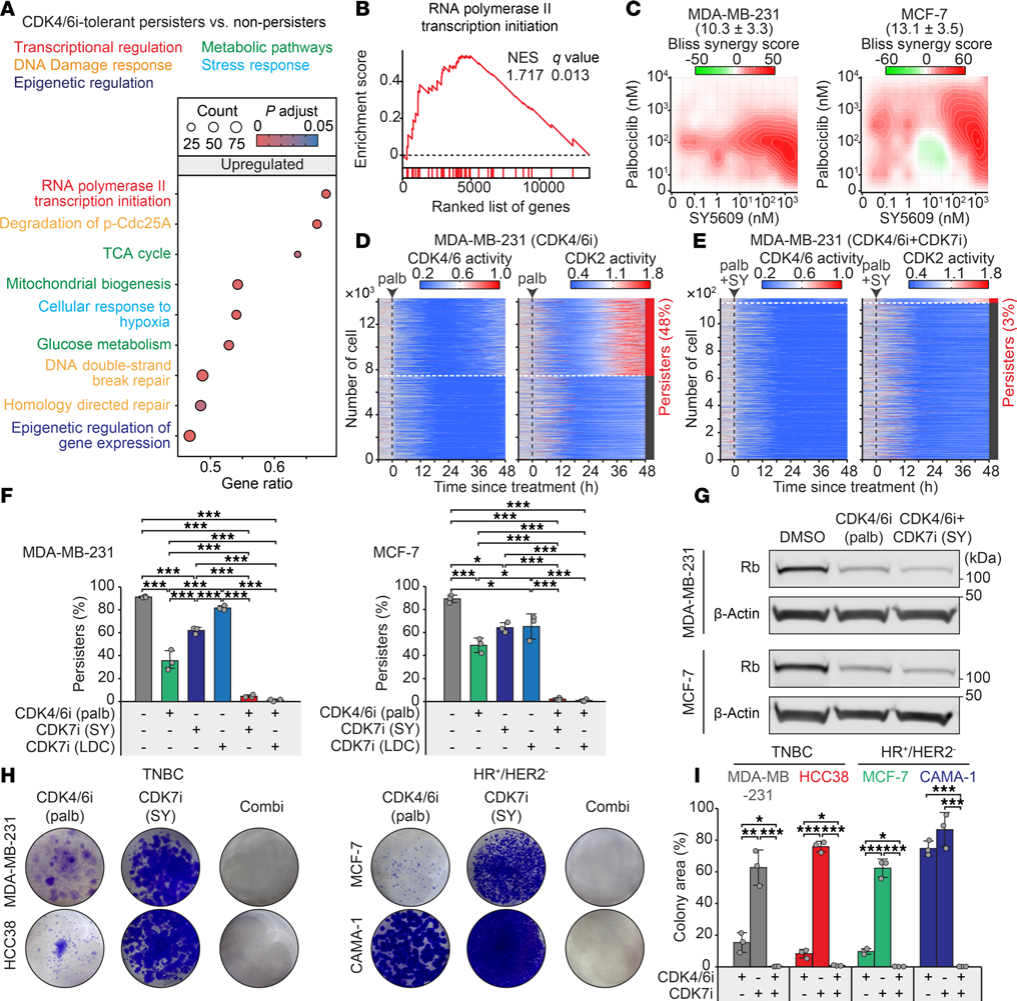
Synergistic inhibition of persister cell development and resistance by combined CDK4/6i and CDK7i. (**A**) Dot plot of enriched reactome gene sets in persister versus non-persister MDA-MB-231 cells after 14 days of palbociclib (1 μM) treatment. Significant enriched pathways were defined as adjusted *P* < 0.05 and FDR < 0.25. Dot size indicates gene count, and color indicates adjusted *P* values. (**B**) GSEA plot showing increased RNA polymerase II signaling in persister cells relative to non-persister cells. Normalized enrichment score (NES) and *q* values are shown. (**C**) Synergy analysis of palbociclib and SY5609. Synergy scores ± 95% CIs were calculated using the Bliss independence model (*n* = 3 biological replicates). (**D** and **E**) Heatmaps of single-cell CDK4/6 and CDK2 activity traces in MDA-MB-231 cells treated with palbociclib (1 μM) alone (**D**) or in combination with SY5609 (50 nM) (**E**). Percentages denote the proportion of persister cells (CDK2 activity > 1.0 for over 4 h during 30–48 h after treatment). (**F**) Quantification of persister cells in MDA-MB-231 and MCF-7 cells across treatment conditions. Data are shown as means ± SD (*n* = 3 biological replicates). *P* values were calculated by 1-way ANOVA with post hoc Tukey’s test (*P ≤ 0.05; ***P ≤ 0.0001). (**G**) Immunoblot analysis of Rb and β-actin in cells treated with DMSO, palbociclib (1 μM), or palbociclib + SY5609 (50 nM) for 48 h. (**H**) Representative clonogenic assay showing colony formation after 28-day treatment with palbociclib (1 μM), SY5609 (50 nM), or their combination (*n* = 3 biological replicates). (**I**) Quantification of colony area (%) across treatments. Data are shown as means ± SD (*n* = 3 biological replicates). *P* values were calculated by 1-way ANOVA with post hoc Tukey’s test (*P ≤ 0.05; **P ≤ 0.001; ***P ≤ 0.0001).

To investigate the signaling pathways underlying CDK4/6i resistance, we isolated persister cells, which drive residual cancer growth and the emergence of drug resistance (39, 40), and performed RNA-Seq. After 14 days of CDK4/6i treatment in MDA-MB-231 cells, we used FACS with a geminin degron to classify persister and non-persister cells. The geminin degron selectively accumulates during the S and G2 phases (41), serving as a marker for proliferating cells. Gene set enrichment analysis (GSEA) revealed distinct upregulated and downregulated pathways in persister cells compared with non-persister cells (Figure 2A and Supplemental Figure 4A). We found that RNA polymerase II–mediated transcription activity was significantly upregulated in persister cells (Figure 2, A and B). Consistent with these findings, our recent studies highlight the pivotal role of transcriptional activity in driving CDK4/6i resistance (19–21). Following alternative Rb inactivation through degradation, breast cancer cells require increased transcriptional activity to amplify low E2F activity, ultimately leading to CDK4/6i resistance. Given that CDK7i primarily targets RNA polymerase II–mediated transcription, we hypothesized that dual inhibition of CDK4/6 and CDK7 could synergistically suppress E2F activity, offering a promising therapeutic strategy to overcome drug resistance.

To test this hypothesis, we performed dose titration experiments with CDK4/6i and CDK7i and observed a synergistic interaction (Figure 2C). We next examined the impact of this combination on persister cell development using MDA-MB-231 and MCF-7 cell lines expressing live-cell sensors for CDK4/6 and CDK2 activities, along with a Cdt1 degron reporter to monitor cell cycle transitions (42, 43). The Cdt1 degron is degraded during the S phase, marking G1/S and S/G2 transitions. This system enabled us to track distinct steps in cell cycle entry and evaluate how cancer cells develop into a persister phenotype under drug treatment (Supplemental Figure 4B). In the absence of treatment, most cells (MDA-MB-231, 89%; MCF-7, 96%) continuously activated both CDK4/6 and CDK2, initiating ongoing proliferation (Supplemental Figure 4C). CDK4/6 inhibition induced robust cell cycle arrest in all cells within 24 h (Figure 2D and Supplemental Figure 4D), confirming that breast cancer cells with an intact Rb/E2F pathway are initially responsive to CDK4/6i. However, a subset of cells (MDA-MB-231, 48%; MCF-7, 45%) acquired a persister phenotype, characterized by CDK2 activation in the absence of high CDK4/6 activity. While CDK7 inhibition alone did not effectively suppress CDK4/6 activity or cell proliferation, its combination with CDK4/6i significantly reduced the emergence of persister cells compared with either monotherapy (Figure 2, E and F, and Supplemental Figure 4, E and F). This combinatorial effect was consistently maintained over a 14-day treatment period (Supplemental Figure 5). Importantly, CDK4/6i reduced Rb levels regardless of CDK7i cotreatment (Figure 2G), suggesting that CDK7i acts downstream to block the transcriptional amplification required for resistance following Rb degradation. Finally, long-term clonogenic assays showed that the CDK4/6i and CDK7i combination significantly inhibited colony formation in both TNBC and HR^+^/HER2^–^ models compared with monotherapies (Figures 2, H and I). Together, these results demonstrate that dual CDK4/6 and CDK7 inhibition effectively prevents the emergence of persister and drug-resistant cells in breast cancer.

### Synergistic suppression of E2F and Myc pathways, along with the upregulation of immune responses, by CDK4/6i and CDK7i treatment.

**Figure 3:**
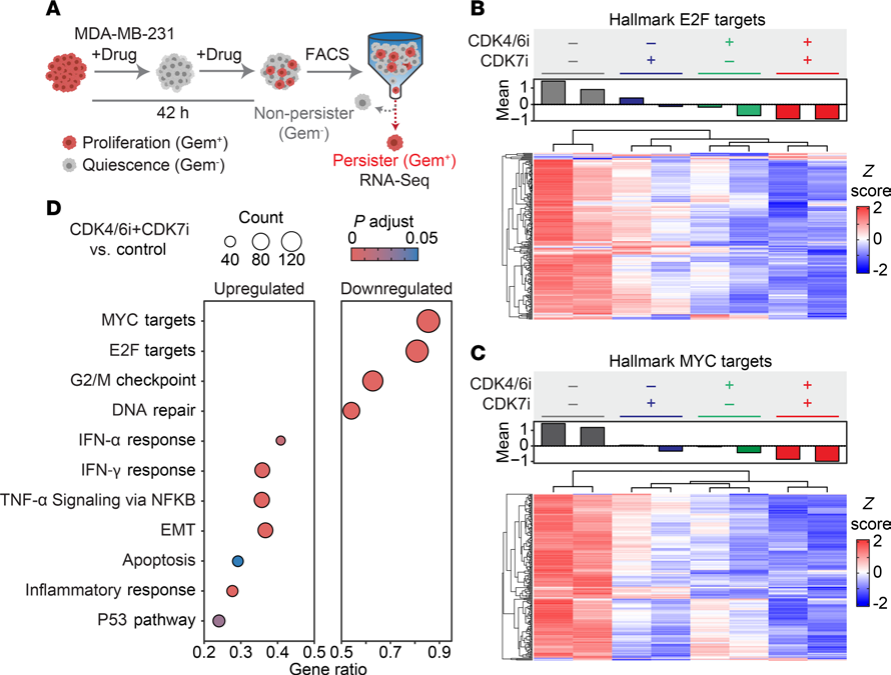
Differential gene expression and pathway modulation in persister cells. (**A**) Schematics of the experimental workflow for isolating persister cells based on geminin degron expression for RNA-Seq analysis. (**B** and **C**) Heatmaps showing the expression levels of E2F (**B**) and Myc (**C**) target genes in persister cells treated with palbociclib (1 μM), SY5609 (50 nM), or their combination. (**D**) Dot plot illustrating significantly enriched or depleted hallmark gene sets in persister cells treated with palbociclib (1 μM) + SY5609 (50 nM) combination compared with untreated controls. Enrichment was determined by adjusted *P* < 0.05 and FDR < 0.25. Dot size indicates gene count, and color indicates adjusted *P* values.

To investigate the molecular effects of combined CDK4/6i and CDK7i therapy in breast cancer, we performed RNA-Seq on persister cells that emerged following drug exposure. Persister cells were isolated 42 h after treatment using FACS based on geminin degron accumulation, with untreated proliferating cells serving as controls (Figure 3A). Gene expression profiles were analyzed using hallmark gene sets from the Molecular Signatures Database (MSigDB), with a focus on E2F and Myc target pathways. As expected, control cells exhibited the highest expression of E2F and Myc target genes, while persister cells treated with the combination therapy showed the most robust suppression of these pathways (Figure 3, B and C). GSEA confirmed that despite active proliferation, the combination treatment significantly downregulated cell cycle–related pathways, including E2F targets, the G2/M checkpoint, and DNA repair mechanisms (Figure 3D). Additionally, we observed upregulation of pathways associated with epithelial-mesenchymal transition and immune responses, including TNF-α and IFN signaling. These changes suggested that the combination treatment may modulate immune cell populations within the tumor microenvironment (TME). Taken together, these findings support the hypothesis that CDK7i suppresses E2F activity following Rb degradation and c-Myc–driven transcriptional amplification. This dual inhibition strategy offers a promising therapeutic approach to prevent or overcome drug resistance in breast cancer.

### CDK4/6i and CDK7i combination therapy in HR^+^/HER2^–^ breast cancer resistant to CDK4/6i and ET.

**Figure 4:**
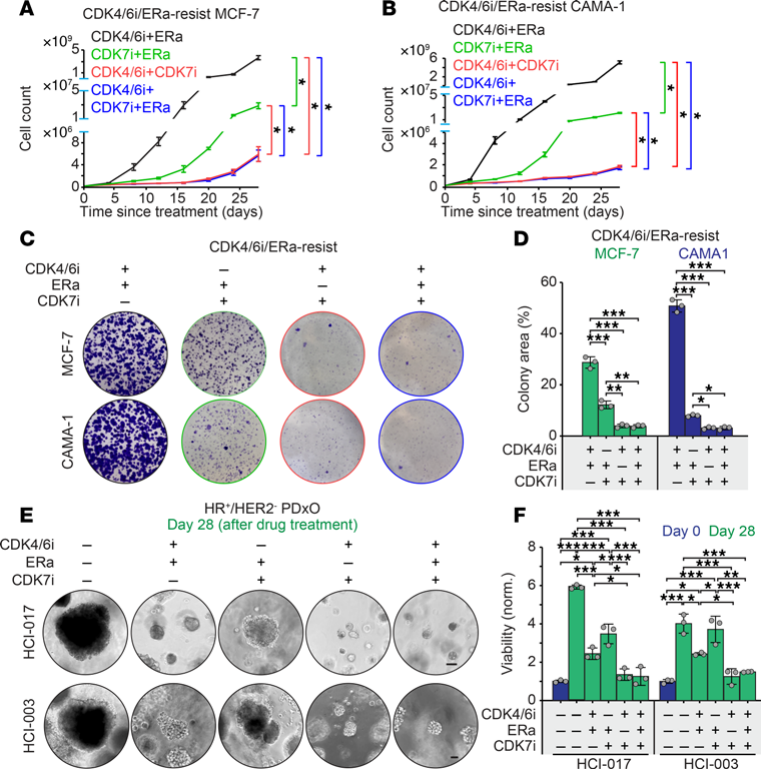
Therapeutic efficacy of CDK4/6i and CDK7i combination in HR^+^/HER2^–^ breast cancer models. (**A** and **B**) Growth curves of palbociclib/fulvestrant-resistant MCF-7 (**A**) and CAMA-1 (**B**) cells treated with the indicated combinations of palbociclib (1 μM), fulvestrant (500 nM), and SY5609 (50 nM). Data are shown as means ± SD (*n* = 3 biological replicates). *P* values were calculated by 2-way ANOVA adjusted based on multiple comparison via Tukey’s test on the final cell count (*P ≤ 0.05). (**C**) Representative clonogenic assay in palbociclib/fulvestrant-resistant MCF-7 and CAMA-1 cells following 28-day treatment with the indicated drugs. Colonies were visualized by crystal violet staining (*n* = 3 biological replicates). (**D**) Quantification of colony area (%) across treatments. Data are shown as means ± SD (*n* = 3 biological replicates). *P* values were calculated by 1-way ANOVA with post hoc Tukey’s test (*P ≤ 0.05; **P ≤ 0.001; ***P ≤ 0.0001). (**E**) Representative bright-field images of HR^+^/HER2^–^ PDxOs (HCI-017 and HCI-003) treated with the indicated combinations of palbociclib (1 μM), fulvestrant (500 nM), and SY5609 (50 nM) at day 28 (*n* = 3 biological replicates). Scale bar: 100 μm. (**F**) Quantification of organoid viability at days 0 and 28. Data are shown as mean ± SD (*n* = 3 biological replicates). *P* values were calculated by 1-way ANOVA with post hoc Tukey’s test (*P ≤ 0.05; **P ≤ 0.001; ***P ≤ 0.0001).

We evaluated the therapeutic potential of CDK7 inhibition for HR^+^/HER2^–^ breast cancer models that had developed resistance to both CDK4/6i and ET. Based on our mechanistic model, both ET and CDK7i suppress the growth of CDK4/6i-resistant tumors by targeting c-Myc–mediated amplification of E2F activity. Therefore, we hypothesized that adding ET to the CDK4/6i and CDK7i combination would not provide additional therapeutic benefit. To test this, we employed MCF-7 and CAMA-1 cell lines resistant to both palbociclib and the estrogen receptor antagonist (ERa) fulvestrant. These resistant cells were treated with various combinations: CDK4/6i+ERa continuously or switching to CDK7i+ERa, CDK4/6i+CDK7i, or CDK4/6i+CDK7i+ERa. Among these, the combination of CDK4/6i and CDK7i most effectively suppressed cell growth, and the addition of ERa did not yield further benefit (Figure 4, A and B). Clonogenic assays confirmed that dual CDK4/6 and CDK7 inhibition significantly reduced colony formation, with no additional effect from ERa cotreatment (Figure 4, C and D). We extended these findings using 2 HR^+^/HER2^–^ patient-derived xenograft organoids (PDxOs). Similarly, CDK4/6i and CDK7i cotreatment significantly inhibited organoid growth, again without further enhancement from ERa (Figure 4, E and F, and Supplemental Figure 6A). Collectively, these results suggest that dual CDK4/6 and CDK7 inhibition, independent of continued ET, represents a promising therapeutic strategy for HR^+^/HER2^–^ breast cancer patients who have progressed on CDK4/6i and ET.

### CDK4/6i and CDK7i combination as a primary therapeutic strategy in TNBC.

**Figure 5:**
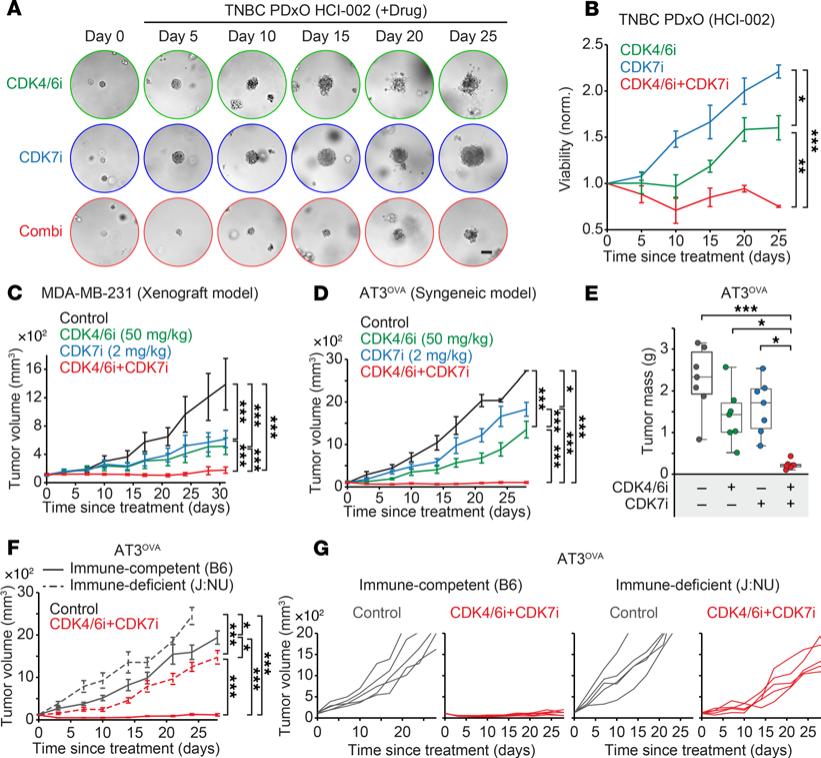
Therapeutic efficacy of CDK4/6i and CDK7i combination in TNBC models. (**A**) Representative bright-field images of TNBC PDxO (HCI-002) treated with palbociclib (1 μM), SY5609 (50 nM), or their combination over time (*n* = 3 biological replicates). Scale bar: 100 μm. (**B**) Growth curve of PDxO (HCI-002) viability under indicated treatments. Data are shown as means ± SD (*n* = 3 biological replicates). *P* values were calculated by 2-way ANOVA with post hoc Tukey’s test (*P ≤ 0.05; **P ≤0.001; ***P ≤ 0.0001). (**C** and **D**) Tumor growth curves for MDA-MB-231 xenograft (**C**) and AT3^OVA^ syngeneic (**D**) mouse models treated with vehicle, palbociclib (50 mg/kg), SY5609 (2 mg/kg), or the combination. Data are shown as means ± SEM (**C**: *n* = 12 mice for CDK4/6i+CDK7i, 8 mice for other groups; **D**: *n* = 7 mice/group). *P* values were calculated by 2-way ANOVA (**C**) or a mixed-effect model (**D**) with post hoc Tukey’s test (*P ≤ 0.05; ***P ≤ 0.0001). (**E**) Box plot of tumor mass for AT3^OVA^ tumors. The middle line indicates the median, with box edges representing interquartile ranges (*n* = 7 mice/group). *P* values were calculated by 1-way ANOVA with post hoc Tukey’s test (*P ≤ 0.05; ***P ≤ 0.0001). (**F** and **G**) Tumor growth curves for AT3^OVA^ model in immunocompetent (C57BL/6J) or -deficient (J:NU) mice. Mice were treated with either vehicle or a combination of palbociclib (50 mg/kg) and SY5609 (2 mg/kg). Data are presented as mean ± SEM (*n* = 5 mice/group) (**F**). *P* values were calculated by a mixed-effect model with post hoc Tukey’s test (*P ≤ 0.05; **P ≤ 0.001; ***P ≤ 0.0001).

We next assessed the efficacy of combining CDK4/6i and CDK7i as a primary therapeutic approach in TNBC models. In 2 TNBC PDxOs, the combination therapy significantly suppressed tumor growth compared with either monotherapy (Figure 5, A and B, and Supplemental Figure 6, B and C). While tumors treated with single agents developed resistance over time, the combination maintained a sustained antitumor effect throughout the 25-day treatment period. To examine the therapeutic potential of CDK4/6i and CDK7i in vivo, we established xenograft and syngeneic TNBC models by orthotopically injecting human MDA-MB-231 cells into immunodeficient J:NU mice and mouse AT3^OVA^ cells into immunocompetent C57BL/6J mice. Once tumors reached an average volume of approximately 100 mm³, mice were treated with CDK4/6i (palbociclib; 50 mg/kg), CDK7i (SY5609; 2 mg/kg), or their combination. Although monotherapies delayed tumor progression, resistance emerged in both models (Figure 5, C–E, and Supplemental Figure 6, D–H). In contrast, combination therapy induced durable tumor suppression across both models. Notably, tumor regression was greater in the immunocompetent syngeneic model compared with the immunodeficient xenograft model. While this may be partly attributed to the more aggressive tumor growth kinetics in the syngeneic model, it also suggests that immune components contribute to the enhanced therapeutic response. To directly test this, we compared the efficacy of the CDK4/6i and CDK7i combination in AT3^OVA^ tumors established in immunodeficient and immunocompetent mice. Combination therapy achieved significantly greater tumor suppression in the presence of an intact immune system (Figure 5, F and G), indicating that the therapeutic benefit involves both tumor-intrinsic and immune-mediated mechanisms.

The combination therapy was well tolerated, with no observed mortality, significant weight loss, or histological abnormalities in the kidney, liver, heart, or lung (Supplemental Figure 7, A and B). Hematological analyses revealed no significant changes in WBC, RBC, or lymphocyte counts, although a modest reduction in neutrophils was observed (Supplemental Figure 7C). Plasma levels of liver enzymes alanine aminotransferase and aspartate aminotransferase remained within normal ranges (Supplemental Figure 7D), indicating no overt hepatotoxicity. Together, these findings demonstrate that the CDK4/6i and CDK7i combination exerts potent antitumor activity in TNBC by concurrently targeting tumor-intrinsic pathways and engaging the immune microenvironment, without inducing systemic toxicity.

### Enhanced antitumor immunity by combined CDK4/6 and CDK7 inhibition.

**Figure 6:**
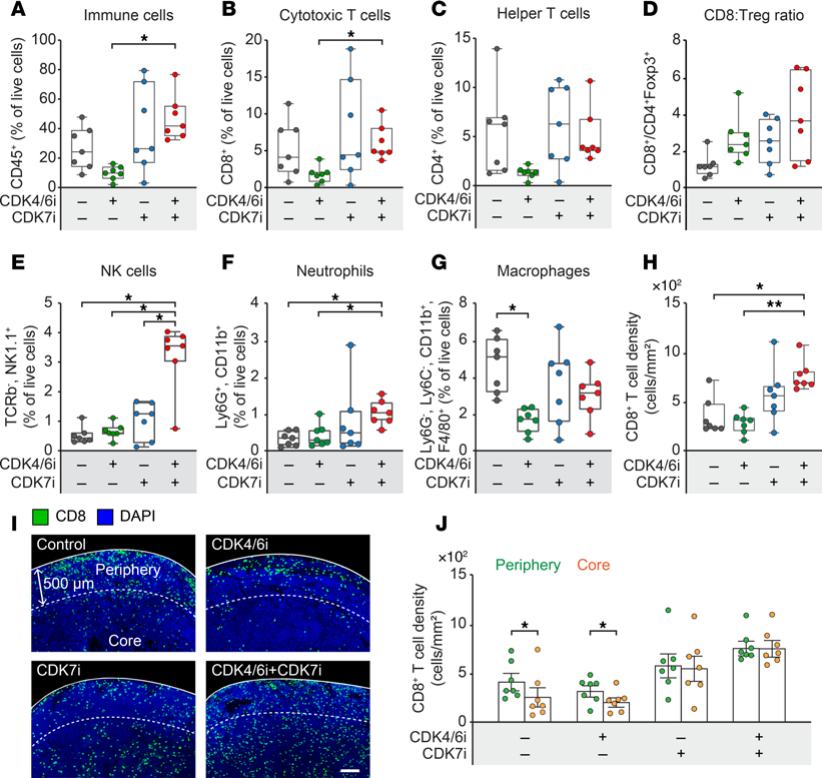
Enhanced antitumor immunity by CDK4/6i and CDK7i treatment. (**A**−**G**) Flow cytometric quantification of immune cell populations in AT3^OVA^ tumors following treatment with vehicle, palbociclib (50 mg/kg), SY5609 (2 mg/kg), or their combination. Total CD45^+^ immune cells (**A**), CD8^+^ T cells (**B**), CD4^+^ T cells (**C**), the ratio of CD8^+^ T cells to Treg cells (**D**), NK cells (**E**), neutrophils (**F**), and macrophages (**G**) are shown. The middle line indicates the median, with box edges representing interquartile ranges (*n* = 7 mice/group). *P* values were calculated by 1-way ANOVA with multiple-comparison Dunnett’s test (*P ≤ 0.05). (**H**) CD8^+^ T cell density per mm^2^ (*n* = 7 mice/group). *P* values were calculated by 1-way ANOVA with multiple-comparison Dunnett’s test (*P ≤ 0.05; **P ≤ 0.001). (**I**) Immunofluorescence images showing spatial distribution of CD8^+^ T cells in the tumor periphery and core. Scale bar: 200 μm. (**J**) Quantification of CD8^+^ T cell density in the tumor periphery and core. Data are shown as means ± SEM (*n* = 7 mice/group). *P* values were calculated by paired *t* test (*P ≤ 0.05).

To investigate the immunomodulatory effects of dual CDK4/6 and CDK7 inhibition, we analyzed the TME in the AT3^OVA^ syngeneic mouse model. Flow cytometric analysis of tumors after 28 days of treatment revealed alterations in immune cell composition. CDK4/6i monotherapy significantly reduced total immune cell infiltration (CD45^+^ cells), likely due to hematopoietic toxicity associated with CDK4/6 inhibition (Figure 6A). However, cotreatment with CDK7i restored and significantly increased CD45^+^ immune cell infiltration compared with both the control and CDK4/6i-alone groups. This effect extended to key T cell populations: combination therapy significantly enhanced infiltration of cytotoxic (CD8^+^) and helper (CD4^+^) T cells relative to CDK4/6i monotherapy (Figure 6, B and C). Moreover, the ratio of CD8^+^ T cells to Treg cells, an indicator of enhanced cytotoxic T cell activity, was elevated following combination treatment (Figure 6D). The combination therapy also increased the abundance of NK cells and neutrophils while reducing macrophage infiltration (Figure 6, E–G). Given the importance of CD8^+^ T cell localization for antitumor efficacy (44–46), we examined their spatial distribution across whole tumor sections. CD8^+^ T cell density was significantly increased in tumors from the combination group (Figure 6H). In control tumors, CD8^+^ T cells were largely restricted to the tumor periphery (within 500 μm of tumor margins), indicative of a T cell exclusion phenotype (45) (Figure 6, I and J). CDK7i, either alone or in combination, reversed this exclusion, promoting CD8^+^ T cell infiltration into the tumor core. These results suggest that dual CDK4/6 and CDK7 inhibition not only suppresses tumor cell proliferation but also reprograms the TME to support effective antitumor immunity.

**Figure 7:**
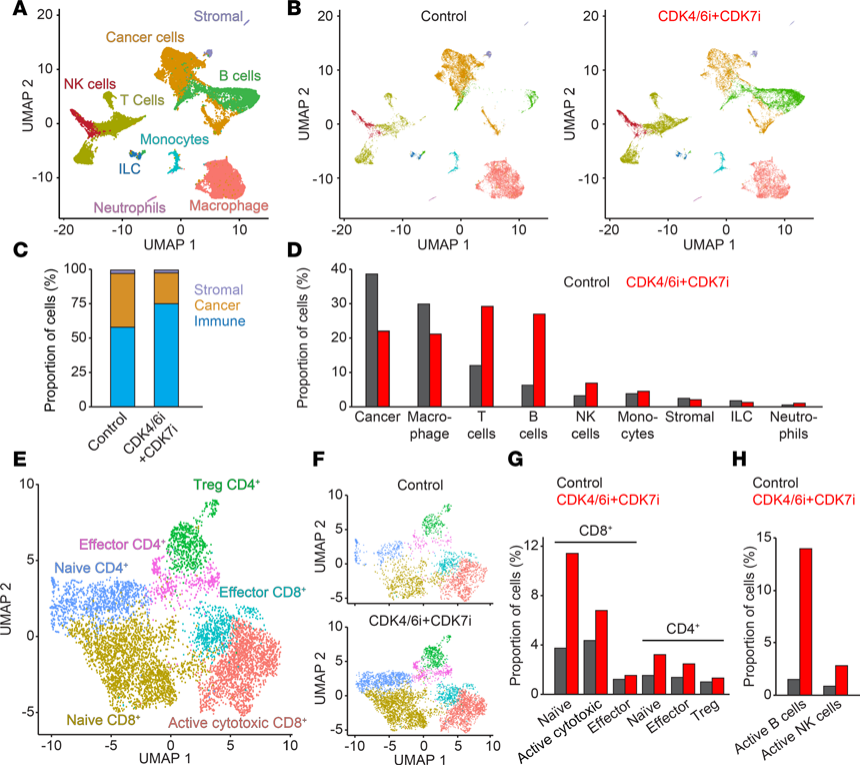
Immune cell dynamics in the TME following CDK4/6i and CDK7i treatment. (**A** and **B**) UMAP plots of single-cell transcriptomes from AT3^OVA^ tumors in total (**A**), control (**B**, left), and combination-treated (50 mg/kg palbociclib and 2 mg/kg SY5609) (**B**, right) groups (*n* = 2 mice/group). (**C**) Stacked bar plot showing relative proportions of immune, cancer, and stromal cells in control and combination groups. (**D**) Quantification of major immune cell populations in control and combination groups. (**E** and **F**) UMAP plots of T cell subpopulations in total (**E**), control (**F**, top), and combination-treated (**F**, bottom) tumors. (**G**) Quantification of CD8^+^ and CD4^+^ T cell subtypes in control and combination groups. (**H**) Quantification of activated B (*CD69*^+^ or *CD83*^+^) and NK (*GZMB*^+^ or *PRFN1*^+^) cells in control and combination groups.

To further elucidate the mechanisms underlying these immune changes, we performed single-cell RNA-Seq of the TME. Transcriptomic profiling of 17,163 control and 20,096 drug-treated cells revealed distinct immune and stromal populations using Uniform Manifold Approximation and Projection–based (UMAP-based) clustering (Figure 7A). Cell identities were annotated based on canonical markers: *EPCAM* and *NFIB* (cancer cells), *CD3D* with *CD8A* and *CD4* (T cells and innate lymphoid cells), *NCR1* (NK cells), *CD68* (macrophages), *CCR2* (monocytes), *CD79A* (B cells), *S100A8* (neutrophils), and *SPARC* (stromal cells) (Supplemental Figure 8A). Combination therapy increased the frequency of T cells, B cells, and NK cells while reducing macrophages (Figure 7, B–D), indicating enhanced immune activation. High-resolution clustering further revealed increased proportions of naive and activated cytotoxic CD8^+^ T cells, as well as naive and effector CD4^+^ T cells (Figure 7, E–G, and Supplemental Figure 8B). The frequency of both immature and mature B and NK cells, including activated subsets, was also elevated in the combination group (Figure 7H and Supplemental Figure 8, C and D), highlighting the capacity of CDK coinhibition to broadly enhance immune-mediated antitumor responses.

**Figure 8:**
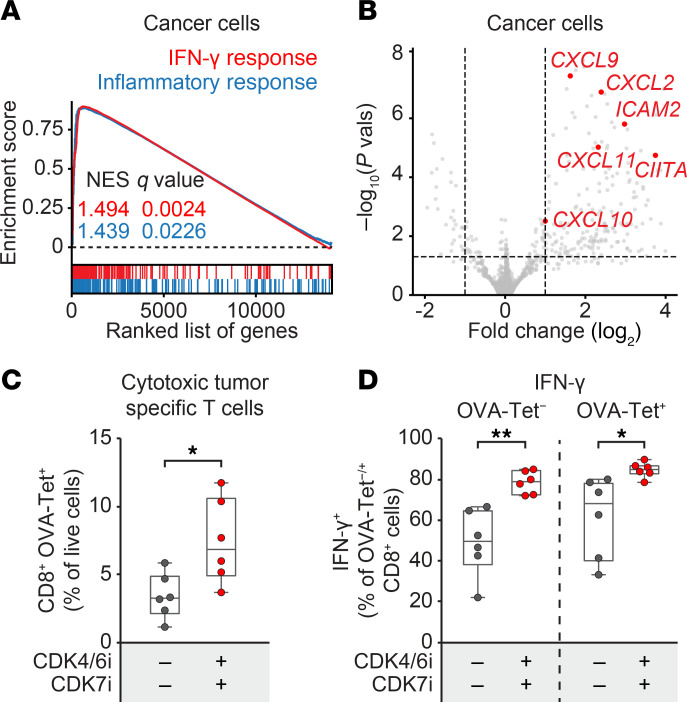
CDK4/6i and CDK7i combination enhances immune-modulatory signaling and tumor antigen–specific T cell responses. (**A**) GSEA plot showing upregulation of IFN-γ and inflammatory response pathways in cancer cells isolated from AT3^OVA^ tumors treated in vivo with a combination of palbociclib (50 mg/kg) and SY5609 (2 mg/kg). Normalized enrichment score (NES) and *q* values are indicated for each pathway. (**B**) Volcano plot of differentially expressed genes in cancer cells following in vivo combination therapy, highlighting key immune-modulatory genes. (**C**) Flow cytometric quantification of OVA-specific CD8^+^ T cells in AT3^OVA^ tumors following treatment. (**D**) Flow cytometric quantification of IFN-γ^+^–producing CD8^+^ T cells in AT3^OVA^ tumors, stratified by OVA tetramer expression. (**C** and **D**) The middle line indicates the median, with box edges representing interquartile ranges (*n* = 6 mice/group). *P* values were calculated by unpaired *t* test (*P ≤ 0.05; **P ≤ 0.001).

GSEA of cancer cell transcriptomes revealed significant upregulation of IFN-γ and inflammatory signaling pathways following combination treatment (Figure 8A). Macrophages showed similar activation of IFN-γ, TNF-α, and inflammatory signaling pathways (Supplemental Figure 9A). In addition, we observed elevated expression of immune-stimulatory cytokines and chemokines in cancer cells, including *CXCL2*, *CXCL9*, *CXCL10*, *CXCL11*, *CIITA*, and *ICAM2* (Figure 8B). These genes are critical for recruiting and activating immune effector cells within the TME (47–49), potentially reinforcing robust antitumor immunity.

To directly assess tumor antigen–specific responses, we conducted a tetramer assay using an H-2K^b^–SIINFEKL MHC class I tetramer that detects CD8^+^ T cells recognizing the OVA peptide expressed by AT3^OVA^ tumors. Flow cytometry analysis revealed a significant increase in both total and OVA-specific CD8^+^ T cells following combination treatment (Figure 8C and Supplemental Figure 9B). OVA-specific CD8^+^ T cells exhibited higher IL-2 expression than nonspecific CD8^+^ T cells (Supplemental Figure 9C), indicating enhanced activation and proliferative potential. Moreover, combination therapy significantly increased IFN-γ production in both OVA-specific and nonspecific CD8^+^ T cells, while TNF-α levels remained unchanged (Figure 8D and Supplemental Figure 9D), suggesting a functionally enhanced cytotoxic T cell response. Collectively, these findings demonstrate that combined CDK4/6 and CDK7 coinhibition reprograms the TME by boosting immune infiltration, reversing immune exclusion, and activating tumor-specific cytotoxic T cells, thereby promoting a robust and durable antitumor immune response.

## Discussion

Our study demonstrates that CDK7 inhibition primarily targets RNA polymerase II activity, thereby attenuating mRNA transcription. Building upon our previous findings implicating transcriptional activity in non-canonical cell cycle entry and CDK4/6i resistance (19, 20), we show that coinhibition of CDK4/6 with CDK7 enhances antitumor responses in both HR^+^/HER2^–^ breast cancer and TNBC models. Transcriptomic analyses further revealed that this combination therapy activates immune-related pathways in cancer cells, including IFN-γ signaling, TNF-α signaling, and cytokine production pathways, which collectively stimulate antitumor immunity within the TME. This immune activation is accompanied by increased infiltration of CD8^+^ and CD4^+^ T cells, B cells, and NK cells, key immune subsets required for effective tumor clearance (50–52). Together, these findings suggest that dual CDK4/6 and CDK7 inhibition not only suppresses tumor-intrinsic proliferation but also reprograms the TME to support immune-mediated tumor elimination, offering a promising therapeutic strategy for breast cancers with an intact Rb/E2F pathway.

This combination shows promise for HR^+^/HER2^–^ breast cancer that has progressed following CDK4/6i and ET. Notably, the addition of ET to the CDK4/6i and CDK7i combination did not provide additional therapeutic benefit, likely due to convergent mechanisms targeting c-Myc–mediated amplification of E2F activity. In TNBC, where effective targeted therapies remain limited, the CDK4/6i and CDK7i combination demonstrated robust antitumor activity when used as a primary treatment. Both xenograft and syngeneic models showed durable tumor suppression, with greater efficacy observed in immunocompetent mice. Single-cell RNA-Seq confirmed enhanced recruitment of T cells, B cells, and NK cells to the TME, supporting an immunostimulatory mechanism of action. These findings provide strong preclinical rationale for evaluating CDK4/6i and CDK7i combination therapy in clinical trials for breast cancer.

Our data support the notion that CDK7i selectively inhibits RNA polymerase II–dependent transcription, consistent with prior studies that identified CDK7i as a therapeutic vulnerability in Myc-driven tumors (53, 54). While higher doses of CDK7i can inhibit additional CDKs and enhance antiproliferative effects, they also increase the risk of systemic toxicity due to broad transcriptional suppression, highlighting the need for careful dose optimization. Thus, the therapeutic window for CDK7 inhibition will likely be defined by balancing efficacy against transcriptional toxicity.

In conclusion, our findings establish dual CDK4/6 and CDK7 inhibition as a rational and effective therapeutic strategy for HR^+^/HER2^–^ and TNBC subtypes and potentially for other malignancies with an intact Rb/E2F pathway. This work advances our understanding of CDK-targeted therapies and underscores the potential addition of CDK7i to overcome CDK4/6i resistance and improve clinical outcomes in breast cancer, warranting further clinical investigation.

## Methods

### Sex as a biological variable.

All in vivo studies were performed using female mice, as the disease being modeled — breast cancer — primarily affects females.

### Cell lines.

MCF-7 (HTB-22), MDA-MB-231 (CRM-HTB-26), and CAMA-1 (HTB-21) cell lines were obtained from ATCC and cultured in DMEM (Genesee Scientific; 25-500) supplemented with 10% FBS (Gibco; 10437-028). AT3^OVA^ cells were cultured in DMEM with 10% FBS. HCC38 cells were cultured in RPMI-1640 medium (Sigma-Aldrich; R8758) supplemented with 10% FBS, 10 mM HEPES (Sigma-Aldrich; H3537), and 1 mM sodium pyruvate (Gibco; 11360-070). All cells were maintained at 37°C in a humidified incubator with 5% CO_2_. All cell lines tested negative for mycoplasma contamination.

### Plasmid generation.

Live-cell biosensors for CDK2 and CDK4/6 activity and cell cycle phase monitoring were generated as previously described and are available from Addgene (19, 20, 32). The following constructs were used: pLenti-DHB (aa 994–1,087)-mVenus-p2a-mCherry-Rb (aa 886–928) (Addgene; 126679), pLenti-H2B-iRFP670-p2a-mCerulean-Cdt1 (aa 1–100) (Addgene; 223965), and pLenti-H2B-iRFP670-p2a-mCerulean-Geminin (aa 1−110) (Addgene; 223959).

### Cell line generation.

Stable cell lines were generated using lentiviral transduction. Lentiviruses were produced in HEK293T cells cotransfected with pCMV-VSV-G (Addgene; 8454), pRSV-rev (Addgene; 12253), and pMDLg/pRRE (Addgene; 12251) using polyethylenimine transfection reagent. Viral supernatants were collected for 3 days after transfection, centrifuged at 500*g* for 5 min, and filtered through a 0.45 μm membrane (Millipore; SLHA033SB). The filtrates were concentrated using Amicon Ultra-15 centrifugal filters with a 100 kDa cutoff (Millipore; UFC910024) and stored at −80°C. Target cells were transduced with lentivirus in the presence of 5 μg/mL polybrene (Sigma-Aldrich; TR-1003-G). Antibiotic selection was initiated 2 days after infection, based on the selectable marker associated with each construct: puromycin (1 μg/mL; InvivoGen; ant-pr-1), blasticidin (10 μg/mL; InvivoGen; ant-bl-1), or neomycin (400 μg/mL; Thermo Fisher Scientific; BP673-5). Palbociclib/fulvestrant-resistant MCF-7 and CAMA-1 cells were generated as previously described (21). These cells were continuously maintained in media containing 1 μM palbociclib and 500 nM fulvestrant, with drug-containing media replenished every 2–3 days. Resistance was validated by determining the IC50 value.

### Drug, chemicals, and antibodies.

EdU (catalog 900584), crystal violet (catalog C0775), hydrocortisone (catalog H0888), and *N*-acetyl-l-cysteine (NAC) (catalog A9165) were obtained from Sigma-Aldrich. EU (catalog 1261) was obtained from Click Chemistry Tools. Palbociclib hydrochloride (HY-50767C), LDC4297 (HY-12653), SY5609 (HY-138293), and Y-27632 (HY-10071) were sourced from MedChem Express; fulvestrant (S1191) was purchased from Selleck Chemistry; and tagtociclib (CT-PF0710) was purchased from Chemietek. Growth factor–reduced Matrigel (catalog 354230) was obtained from Corning. Additional reagents included TRIzol LS (catalog 10296010) and GlycoBlue (catalog AM9515) from Invitrogen, chloroform (catalog J67241K2) from Thermo Fisher Scientific, GlutaMAX (catalog 35050061) from Gibco, PF-06873600 (catalog 35502) from Cayman Chemical, gentamicin (catalog 25-533) from Genesee Scientific, recombinant human EGF (catalog HZ-1326) from ProteinTech, FGF2 (catalog 4114-TC-01M) from R&D Systems, and CellTiter-Glo (catalog G9682) from Promega. Primary antibodies used for immunoblotting included anti-Rb (clone 4H1; catalog 9309), anti-Rpb1 (clone D8L4Y; catalog 14958), anti–phospho-Rpb1 CTD (Ser2) (clone E1Z3G; catalog 13499), anti–phospho-Rpb1 CTD (Ser5) (clone D9NGI; catalog 13523), anti–phospho-Rpb1 CTD (Ser7) (clone E2B6W; catalog 13780), and anti–β-actin (clone 8H10D10; catalog 3700), all from Cell Signaling Technology. Intercept (TBS) blocking buffer (catalog 927-60001), goat anti-mouse IRDye 800CW (catalog 926-32210), and goat anti-rabbit IRDye 680RD (catalog 926-68071) were obtained from LI-COR Biosciences. Fluorophore-conjugated antibodies used for flow cytometry are listed in Supplemental Table 1.

### Organoid culture.

Breast cancer PDxOs (HCI-002, HCI-003, HCI-017, and HCI-041) were obtained from the Huntsman Cancer Institute and cultured according to published protocols (55). PDxOs were embedded in 200 μL of growth factor–reduced Matrigel over a 50 μL Matrigel base layer in multiwell plates. TNBC PDxOs were maintained in PDxO base medium composed of advanced DMEM/F12 (Gibco; 12634028) supplemented with 5% FBS, 10 mM HEPES, 1× GlutaMAX, 1 μg/mL hydrocortisone, 50 μg/mL gentamicin, and 10 ng/mL human EGF with 10 μM Y-27632 freshly added. HR^+^/HER2^–^ PDxOs were cultured in the same base medium supplemented with 10 μM Y-27632, 100 ng/mL FGF2, and 1 mM NAC. Cultures were maintained at 37°C in a humidified atmosphere with 5% CO_2_, and medium was refreshed every 3−4 days. Once organoids reached maturity, they were passaged and reseeded at a density of 2 × 10^5^ cells per Matrigel dome in 6-well plates.

### Organoid viability assay.

To evaluate PDxO viability, organoids were seeded at 2 × 10^4^ cells per dome in 90 μL of Matrigel atop a 10 μL Matrigel base layer in 24-well plates (Fisher Scientific; FB012929). Drug treatments were initiated 7 days after plating, and media were replaced every 3−4 days. Following the treatment period, plates were brought to room temperature, and media were aspirated. Each well received 100 μL of CellTiter-Glo reagent, and plates were gently shaken for 25 min at room temperature. Luminescence was measured using a SpectraMax i3x plate reader (Molecular Devices) after transferring the lysates to 96-well white opaque plates (Corning; 3917).

### Clonogenic assay.

Cells were seeded at equal densities into 6-well plates (Genesee Scientific; 25-105) and treated with the indicated drugs on the day of plating. Drug-containing media were replaced every 2–3 days for a total duration of 28 days. After treatment, cells were fixed by adding 4% paraformaldehyde (PFA) (Ted Pella; NC1537886) containing 10 mM HEPES in PBS (Sigma-Aldrich; D8537), directly mixed with the culture medium at a 1:1 ratio to achieve a final concentration of 2% PFA. Fixation was carried out at room temperature for 15 min. Cells were then washed with PBS and stained with 500 μL of 0.5% crystal violet solution (prepared in 80% distilled water and 20% methanol) per well. Plates were placed on a shaker for 30 min at room temperature, followed by thorough rinsing with water to remove excess dye. Plates were allowed to air dry overnight. Colony area was quantified using ImageJ (NIH) software.

### Immunofluorescence.

Cells were plated in 96-well glass-bottom plates (Cellvis; P96-1.5P) and treated with the indicated drugs. To assess S phase entry and transcriptional activity, cells were labeled with either EdU (10 μM) or EU (1 mM) and incubated at 37°C for 15 min (EdU: for IC50 measurements), 24 h (EdU: for synergy experiments), or 30 min (EU for transcription measurements) before fixation. Cells were fixed in 2% PFA by mixing 4% PFA (prepared in 10 mM HEPES-PBS) with the culture medium at a 1:1 ratio, incubating for 15 min at room temperature. Cells were then permeabilized in PBS with 0.2% Triton X-100 (Sigma-Aldrich; X100) for 15 min. Click chemistry was used to detect EdU or EU incorporation using Alexa Fluor 647–conjugated detection reagents (Click Chemistry Tools; 1300), following the manufacturer’s protocol. Nuclei were counterstained with Hoechst 33342 (10 μg/mL) (Thermo Fisher Scientific; 62249) for 15 min. After each staining step, cells were washed with PBS and stored in PBS until imaging. For synergy assays, cells were treated with drug combinations for 48 h, with EdU added during the final 24 h. Drug synergy was calculated using the Bliss independence model via SynergyFinder (www.synergyfinder.org) (56).

### Immunoblots.

Cells were seeded in 10 cm dishes and treated with the indicated drugs for the specified durations. Cells were washed with ice-cold PBS and lysed in RIPA buffer supplemented with protease (Thermo Fisher Scientific; 1861279) and phosphatase (Sigma-Aldrich; 4906845001) inhibitor cocktails. Lysates were clarified by centrifugation at 12,000*g* for 10 min at 4°C. Protein concentrations were determined using the Pierce 660 nm Protein Assay Reagent (Thermo Fisher Scientific; 22660). Equal amounts of protein (10–20 μg) were mixed with Laemmli sample buffer containing 2% 2-mercaptoethanol (Bio-Rad; 1610710) and boiled at 95°C for 5 min. Proteins were resolved on either 8% hand-cast SDS-PAGE gels or NuPAGE 4%–12% Bis-Tris precast gels (Invitrogen; NP0321) and transferred to membranes. Membranes were blocked in 5% blocking buffer in TBS for 1 h at room temperature and incubated overnight at 4°C with primary antibodies: anti–β-actin (1:2,000), anti-Rb (1:2,000), anti-Rpb1 (1:1,000), and anti–phospho-Rpb1 CTD (Ser2, Ser5, and Ser7) (each 1:1,000). Blots were washed in TBS-T (0.1% Tween 20), incubated with IRDye-labeled secondary antibodies — goat anti-mouse IRDye 800CW or goat anti-rabbit IRDye 680RD (1:2,000; LI-COR Biosciences) — for 1 h at room temperature and imaged using the LI-COR Odyssey Infrared Imaging System.

### Live- and fixed-cell imaging.

Cells were seeded in a 96-well glass-like polymer bottom plate (Cellvis; P96-1.5P) at densities optimized to maintain 30%–80% confluency throughout the experiment (57). Multicolor fluorescence imaging was performed using an Eclipse Ti2 inverted microscope (Nikon Instruments), equipped with either a ×10 (numerical aperture [NA] 0.45, bin 1) or ×20 (NA 0.75, bin 2) objective. For live-cell imaging, cells were maintained in a humidified chamber (Tokai Hit) at 37°C with 5% CO_2_ and imaged every 12 min. Total light exposure was kept below 400 ms per time point to minimize phototoxicity. Image acquisition and analysis were performed using ImageJ and custom MATLAB scripts.

### Image analysis.

Automated image processing was conducted using MATLAB-based algorithms as previously described (19). In fixed-cell experiments, nuclei were segmented via Hoechst 33342 staining using histogram-based thresholding. In live-cell experiments, nuclear segmentation was achieved by detecting H2B-iRFP670 fluorescence via the Laplacian of Gaussian blob detection method. Flat-field correction was applied to mitigate illumination bias. Cell tracking was performed using the deflection-bridging algorithm. Mitotic events were defined by the appearance of 2 closely adjacent daughter nuclei with combined nuclear intensity comparable to that of the parental nucleus. For kinase translocation reporters, the cytoplasmic-to-nuclear fluorescence ratio was quantified. The cytoplasm was approximated as a ring extending 2–10 μm from the nuclear mask, excluding rings overlapping adjacent nuclei. Nuclear signal was derived from the segmented mask, and cytoplasmic signal was calculated from the median intensity within the defined ring.

### RNA extraction, sequencing, and analysis.

MDA-MB-231 cells expressing mCerulean-tagged geminin (aa 1–110) were treated with the indicated drugs for 42 h or 14 days. Cells were dissociated and sorted by FACS based on mCerulean fluorescence to isolate geminin-positive cells. Immediately after sorting, cells were lysed in TRIzol LS Reagent (Invitrogen; 10296010) and mixed with 0.2 mL of chloroform per 1 mL of TRIzol to induce phase separation. After centrifugation at 12,000*g* for 15 min at 4°C, the aqueous phase was collected and RNA was precipitated by adding 0.5 mL of isopropanol and GlycoBlue (50 μg/mL) (Applied Biosystems; AM9515), followed by overnight incubation at −20°C. Precipitated RNA was pelleted by centrifugation at 12,000*g* for 10 min at 4°C, washed with 75% ethanol, and centrifuged again at 7,500*g* for 5 min. After air-drying, RNA was resuspended in RNase-free water. RNA quality and quantity were assessed using a TapeStation 4200 system (Agilent Technologies). RNA-Seq was performed at the Columbia Genome Center using the Clontech Ultra Low v4 Kit and the Element Aviti platform. Differential expression analysis was conducted with the DESeq2 R package (v1.44.0) (58), applying log2 fold-change shrinkage using the apeglm method (59). GSEA was performed using the clusterProfiler R package (v4.12.6) with hallmark and reactome gene sets from MSigDB. Ranked gene lists were used for input (max gene set size = 500), and statistical significance was assessed with 10,000 permutations. Pathways were considered significantly enriched at *P* < 0.05 and FDR < 0.25.

### In vivo xenograft and syngeneic mouse models.

Female C57BL/6J and J:NU mice (7–8 weeks old) were anesthetized via intraperitoneal injection of ketamine (90 mg/kg) and xylazine (10 mg/kg). AT3^OVA^ cells (5 × 10^5^ cells per mouse in PBS) were orthotopically injected into the abdominal mammary fat pads of both C57BL/6J and J:NU mice. For human xenograft models, MDA-MB-231 cells (2 × 10^6^ cells per mouse) were suspended in Geltrex LDEV-Free Reduced Growth Factor Basement Membrane Matrix (Thermo Fisher Scientific; A1413201) and similarly injected into J:NU mice. Tumor volume was measured biweekly using digital calipers and calculated using the formula: volume = (width^2^ × length) × ½. Drug treatment commenced once tumors reached an average volume of approximately 100 mm³. Mice bearing AT3^OVA^ tumors were administered varying doses of SY5609 (2, 5, 10, or 25 mg/kg in corn oil) or treated with vehicle control (corn oil), palbociclib (50 mg/kg), SY5609 (10 mg/kg), or tagtociclib (50 mg/kg). Mice bearing AT3^OVA^ or MDA-MB-231 tumors received vehicle, palbociclib (50 mg/kg), SY5609 (2 mg/kg), or a combination of palbociclib and SY5609. All treatments were administered once daily by oral gavage. At study endpoint, mice were anesthetized using ketamine (90 mg/kg) and xylazine (10 mg/kg) and subjected to vascular perfusion with either 1% PFA in PBS for immunohistochemistry or ice-cold PBS for flow cytometry sample preparation. Tumors were excised and weighed. Blood was collected 28 days after treatment to evaluate hematological parameters and plasma liver enzyme levels (alanine aminotransferase and aspartate aminotransferase). Blood analyses were performed at the Institute of Comparative Medicine, Columbia University Medical Center using the Heska Element HT5 analyzer for complete blood counts and the Heska Element DC analyzer for biochemical analysis. All animal studies were conducted in accordance with institutional guidelines for the care and use of laboratory animals. Experimental protocols were approved by the Institutional Animal Care and Use Committee at Columbia University Irving Medical Center. Mice were regularly monitored, and ethical endpoints were strictly observed to ensure animal welfare.

### Flow cytometry.

At the experimental endpoint, mice were perfused via the left ventricle with cold PBS for 2 min. Tumors were excised, finely minced with a sterile razor blade, and enzymatically dissociated in a digestion buffer composed of FACS buffer (PBS + 2% FBS), 0.1% collagenase IV (Worthington; LS004188), and 10 U/mL DNase I (Sigma-Aldrich; D4527) at 37°C for 30 min. The resulting single-cell suspensions were filtered through a 70 μm nylon mesh, and RBCs were lysed using 1× RBC Lysis Buffer (eBioscience; 00-4300-54). To evaluate tumor antigen–specific T cell responses, 5 × 10^6^ viable cells were cultured in RPMI medium supplemented with 10% FBS, 1× penicillin/streptomycin (Gibco; 15-140-122), 1× GlutaMAX, 10 mM HEPES, 100 mM sodium pyruvate, 50 μM 2-mercaptoethanol, and 1× MEM nonessential amino acids (Cytiva; SH30238.01). A protein transport inhibitor (Invitrogen; 00-4980-93) was added 6 h before harvesting to allow intracellular cytokine accumulation. Single-cell suspensions were first incubated with anti-mouse CD16/CD32 (clone S17011E) (Fc Block; BioLegend; 156604) to minimize nonspecific antibody binding, followed by staining with Zombie Aqua viability dye (1:1,000; BioLegend; 423101). For detection of tumor-specific CD8^+^ T cells, cells were stained with H-2K^b^–SIINFEKL tetramer conjugated to phycoerythrin (PE) (clone 25-D1.16) (MBL; TS-5001-1C) for 30 min at room temperature, followed by staining with fluorophore-conjugated antibodies: anti-CD8a conjugated to FITC (clone KT15) (1:20; Invitrogen; MA5-16759) and CD45 conjugated to Brilliant Ultraviolet 395 (clone 30-F11) (1:100; BD Biosciences; 564279). For intracellular cytokine analysis, cells were fixed and permeabilized using fixation/permeabilization buffer (eBioscience; 00-5123-43) and subsequently stained with the appropriate fluorophore-conjugated antibodies (TNF-α, Brilliant Violet 650; IL-2, PerCP-Cyanine5.5; and IFN-γ, PE/Cyanine7). All antibodies used are listed in Supplemental Table 1. Samples were acquired on a NovoCyte Penteon flow cytometer (Agilent), and data were analyzed using FlowJo v10.8.1 (BD Biosciences) or NovoExpress v1.6.2 (Agilent).

### Tissue preparation.

Following cardiac perfusion, tumors and organs (heart, kidney, liver, and lung) were collected and fixed in 1% PFA at 4°C for 1 h, then incubated overnight in 30% sucrose at 4°C. Tissues were embedded in optimal cutting temperature compound (Thermo Fisher Scientific; 23-730-571) and sectioned using a cryostat (Leica; CM1850). Tumor sections were cut at 50 μm thickness, and organ sections were cut at 10 μm.

### Immunohistochemistry.

Tumor sections were washed with PBS containing 0.3% Triton X-100 (PBST) and blocked with 5% normal donkey serum (Jackson ImmunoResearch; 017-000-121) for 1 h at room temperature. Sections were incubated overnight at 4°C with rabbit anti-mouse CD8^+^ antibody (clone EPR21769) (1:500; Abcam; ab217344) in blocking buffer. The following day, sections were incubated with Alexa Fluor 594–conjugated donkey anti-rabbit IgG (1:500; Jackson ImmunoResearch; 711-585-152) in PBST for 4 h at room temperature. Nuclei were counterstained with DAPI (Sigma-Aldrich; D9542) for 10 min and mounted using Fluoromount-G (Invitrogen; 00-4958-02).

### H&E staining.

Organ sections were rinsed in distilled water and stained with Mayer’s hematoxylin (Sigma-Aldrich; MHS1-100ML) for 1 min at room temperature. Sections were washed with tap water, treated with Bluing Reagent (Thermo Fisher Scientific; 6769001) for 10 s, and rinsed again. Eosin Y counterstaining (Sigma-Aldrich; 1170811000) was performed for 10 s. Sections were dehydrated through graded ethanol (70%, 96%, and 100%) followed by 2 xylene washes (Fisher Chemical; X3S-4) and mounted with Entellan (Sigma-Aldrich; 1079610100).

### Morphometric analysis.

Images were acquired using an Axio Observer 7 microscope with Apotome2 (Zeiss; ×10 objective, NA 0.45, binning 2). Image analysis was performed using MATLAB as previously described (44). Tumor regions were segmented, and the bwdist function was used to compute distances from tumor margins. CD8^+^ T cell infiltration was quantified as cells/mm² in the tumor periphery (<500 μm from the boundary) and core (>500 μm).

### Single-cell RNA-Seq and analysis.

Tumors were dissociated into single-cell suspensions, resuspended in PBS with 0.4% BSA (Sigma-Aldrich; 9647), and submitted to the Columbia Genome Center for 10X Genomics processing. Gene expression matrices were generated with Cell Ranger and analyzed in R using Seurat v5.1.0 (60). Cells expressing <500 or >5,000 genes, or >10% mitochondrial transcripts, were excluded. Data normalization was performed using SCTransform with mitochondrial regression (61). Canonical correlation analysis was used for batch correction and integration. Dimensionality reduction was performed by principal component analysis, and clustering was conducted with the FindClusters function. Cell types were annotated based on canonical markers. UMAP was used for visualization. Pseudo-bulk differential expression analysis was performed using DESeq2 (58), with effect-size shrinkage using the apeglm method (59). GSEA was conducted using MSigDB with 10,000 permutations; significance was defined as *P* < 0.05 and FDR < 0.25.

### Statistics.

All statistical analyses were conducted using GraphPad Prism v10.3.1. Tests included unpaired or paired 2-tailed Student’s *t* tests, along with 1- or 2-way ANOVA, as specified in the figure legends. *P* value ≤ 0.05 was considered statistically significant. The numbers of biological replicates and sample sizes are detailed in the corresponding figure legends and in the Supporting Data Values file.

### Study approval.

Female C57BL/6J (strain 000664) and J:NU (strain 007850) mice (6–7 weeks old) were obtained from The Jackson Laboratory and housed in a barrier facility with a 12 h light/dark cycle with ad libitum access to food and water. Experimental endpoints were set between 14 and 16 weeks of age. All procedures were conducted in compliance with institutional ethical guidelines and were approved by the Institutional Animal Care and Use Committee at Columbia University Irving Medical Center.

### Data availability.

All data values supporting figures and analyses are included in the Supporting Data Values file. Raw and processed RNA-Seq data have been deposited in the Gene Expression Omnibus under accession number GSE281158. Custom analysis scripts used for processing bulk and single-cell RNA-Seq data are available at https://github.com/Kim-Yang-Lab/CDK4-6i_CDK7i_paper; commit ID: 04a64b7.

## Author contributions

SK and HWY conceptualized the research idea. SK, ES, and HP conducted the investigation. SK, ES, HP, and HWY analyzed the data. SK, ES, and MK provided resources. MK and HWY secured funding for the study. HWY wrote the manuscript and supervised the research. All authors reviewed and edited the manuscript.

## Supplementary Material

Supplemental data

Unedited blot and gel images

Supporting data values

## Version 1 08/12/2025

In-Press Preview

## Version 2 10/15/2025

Electronic publication
