# Supplementary material for: Dual targeting of CDK4/6 and CDK7 augments tumor response and antitumor immunity in breast cancer models
Source: J Clin Invest. 2025 Aug 12;135(20):e188839. doi: 10.1172/JCI188839 (PMC12520673; doi:10.1172/JCI188839)
Supplement: Supplemental data [file jci-135-188839-s113.pdf]

Supplemental Figures

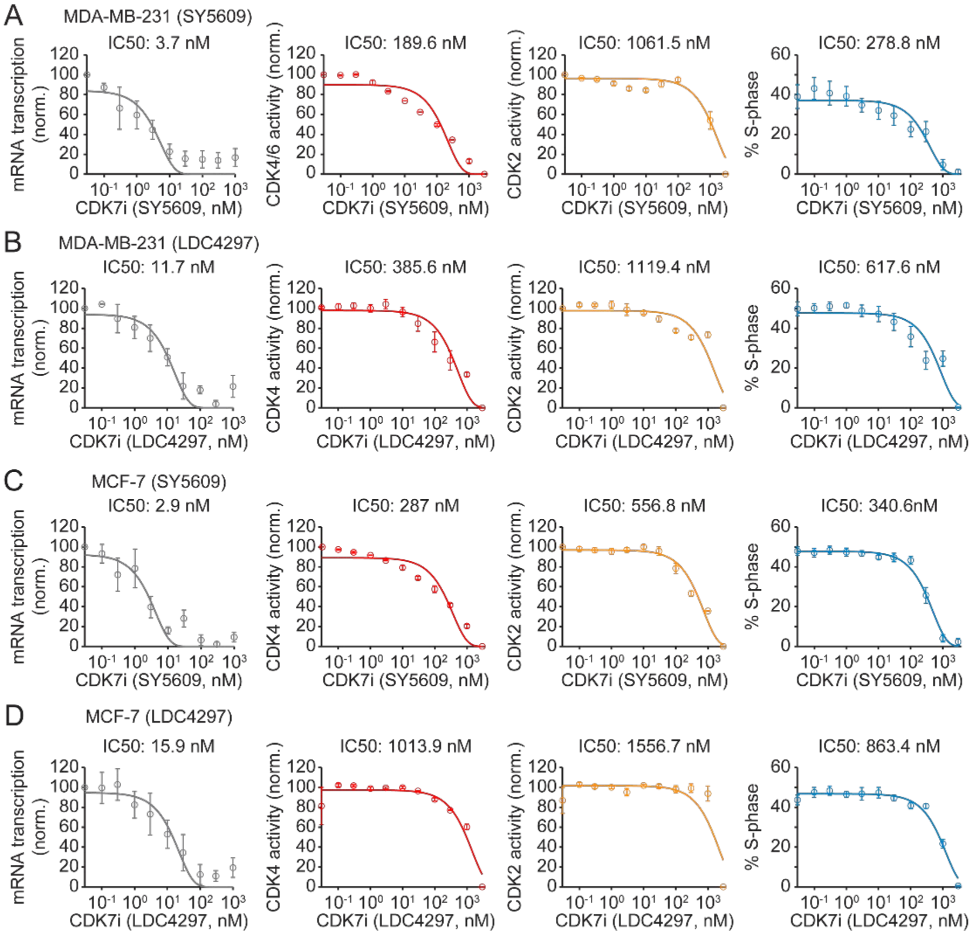

Supplemental Figure 1. Effect of CDK7i on transcription, CDK activity, and cell-cycle progression in breast cancer cells

(**A**−**D**) Dose-response curves of mRNA transcription rates, CDK4/6 and CDK2 activities, and the percentage of S-phase cells in MDA-MB-231 (**A**, **B**) and MCF-7 cells (**C**, **D**) following 2-day treatment with SY5609 (**A**, **C**) or LDC4297 (**B**, **D**). Data are shown as means ± SEM (*n* = 3 biological replicates).

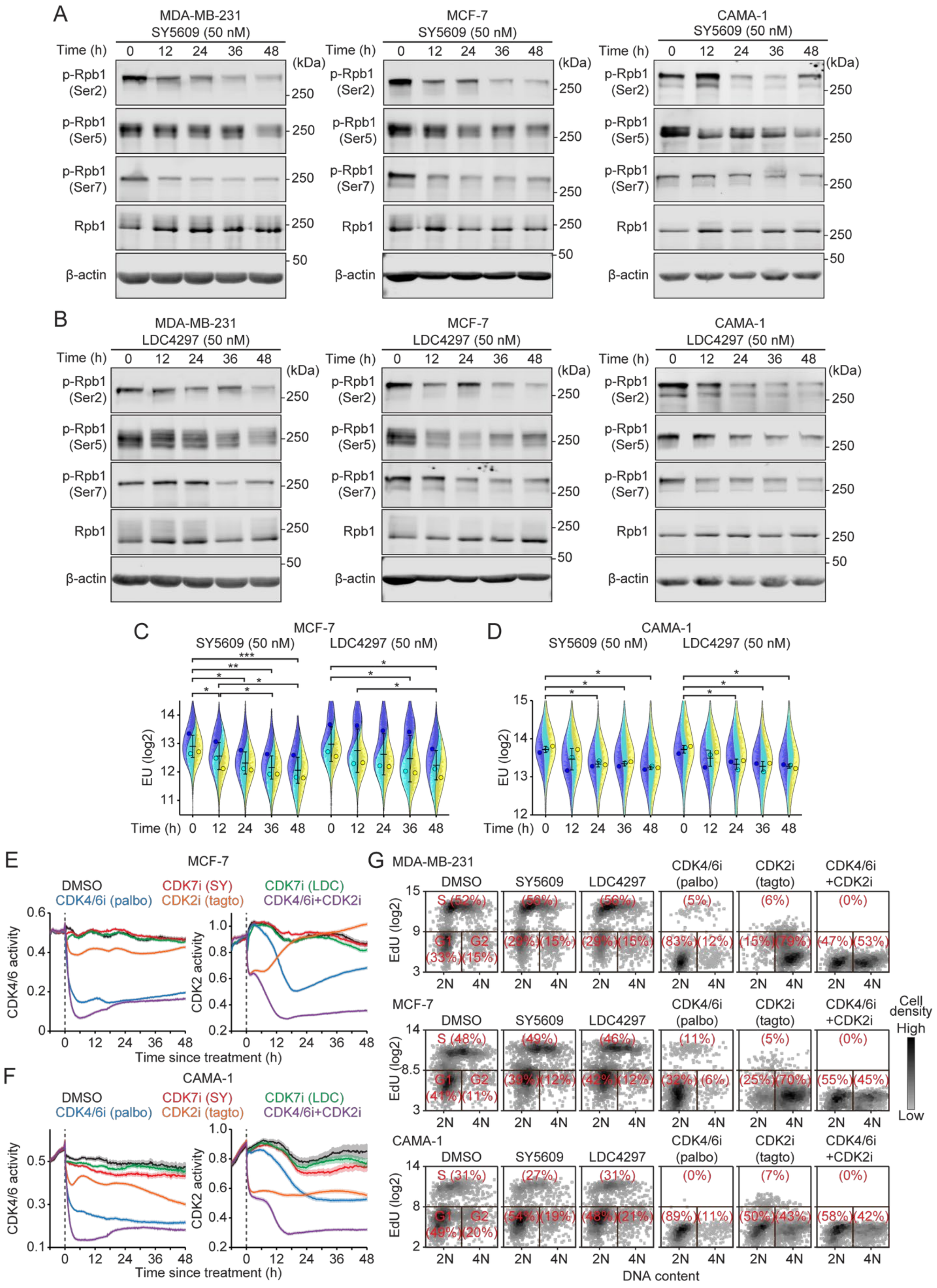

A
MDA-MB-231
SY5609 (50 nM)
MCF-7
SY5609 (50 nM)
CAMA-1
SY5609 (50 nM)
Time (h)
0 12 24 36 48
(kDa)
p-Rpb1 (Ser2)
p-Rpb1 (Ser5)
p-Rpb1 (Ser7)
Rpb1
β-actin
250
50
B
MDA-MB-231
LDC4297 (50 nM)
MCF-7
LDC4297 (50 nM)
CAMA-1
LDC4297 (50 nM)
C
MCF-7
SY5609 (50 nM)
LDC4297 (50 nM)
EU (log2)
D
CAMA-1
E
MCF-7
DMSO
CDK7i (SY)
CDK7i (LDC)
CDK4/6i (palbo)
CDK2i (tagto)
CDK4/6i+CDK2i
CDK4/6 activity
CDK2 activity
Time since treatment (h)
F
CAMA-1
G
MDA-MB-231
MCF-7
CAMA-1
DMSO
SY5609
LDC4297
CDK4/6i (palbo)
CDK2i (tagto)
CDK4/6i +CDK2i
EdU (log2)
DNA content
Cell density
High
Low

Supplemental Figure 2. CDK7 inhibition selectively reduces RNA polymerase II phosphorylation and mRNA transcription without altering cell-cycle CDK activity

(**A** and **B**) Immunoblots showing total RNA Polymerase II (Pol II) and its phosphorylated forms (Ser2, Ser5, Ser7). β-actin was used for loading control. Panels show results from MDA-MB-231, MCF-7, and CAMA-1 cell lines treated SY5609 (50 nM) (**A**) or LDC4297 (50 nM) (**B**).

(**C** and **D**) Violin plots of EU incorporation in MCF-7 (**C**) and CAMA-1 (**D**) cells treated with SY5609 (50 nM) or LDC4297 (50 nM) at the indicated time points. Cells were randomly selected for 1,000 cells per condition in each replicate. Data are shown as means ± SD (n = 3 biological replicates). P values were calculated by two-way ANOVA with post hoc Tukey's test (* $p \leq 0.05$; ** $p \leq 0.001$; *** $p \leq 0.0001$).

(**E** and **F**) Averaged live-cell traces of CDK4/6 and CDK2 activity in MCF-7 (**E**) and CAMA-1 (**F**) cells treated with DMSO, SY5609 (50 nM), LDC4297 (50 nM), palbociclib (1 μM), tagtociclib (5 μM), or palbociclib+tagtociclib. Data are shown as mean ± 95% confidence intervals (n > 1,700 cells/condition).

(**G**) Density scatterplot of DNA content versus EdU incorporation in cells treated with DMSO, SY5609 (50 nM), LDC4297 (50 nM), palbociclib (1 μM), tagtociclib (5 μM), or palbociclib+tagtociclib for 48 h. EdU (10 μM) was added for 15 min prior to fixation (n = 1,500 cells/condition).

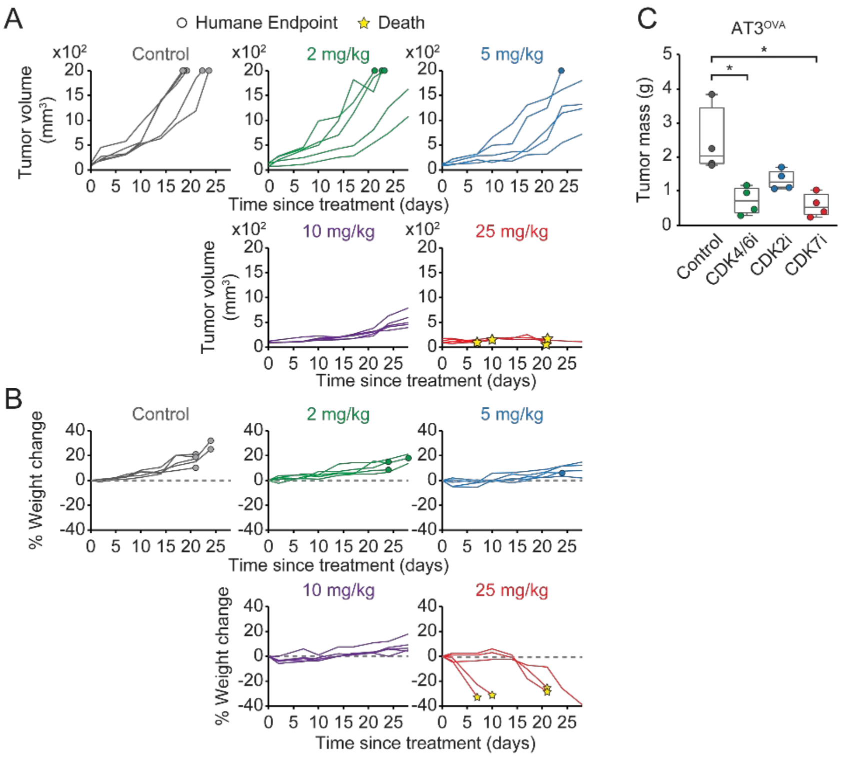

Supplemental Figure 3. Dose-dependent effects of CDK7i on tumor growth and mouse weight (**A** and **B**) Individual tumor growth curves (**A**) and percentage body weight change (**B**) in AT3^{OVA}-bearing C57BL/6J mice treated with escalating doses of SY5609 (0, 2, 5, 10, or 25 mg/kg). Circles indicate the humane endpoint (tumor volume > 2,000 mm^3), and stars denote spontaneous deaths. (**C**) Boxplot of tumor mass at study endpoint across treatment groups. The middle line indicates the median, with box edges representing interquartile ranges (n = 4 mice/group). P values were calculated by one-way ANOVA with post hoc Tukey's test (* $p \leq 0.05$).

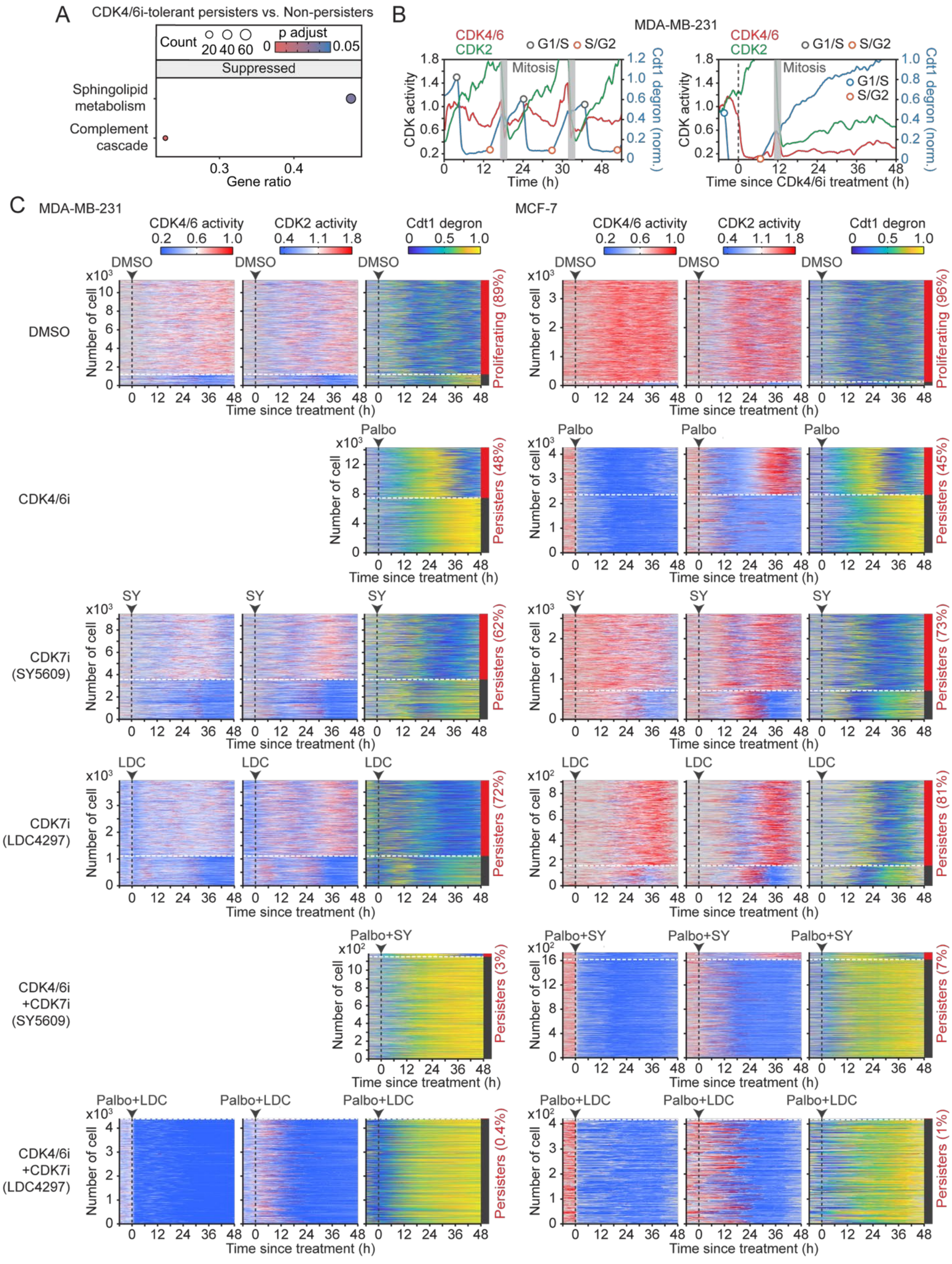

A
CDK4/6i-tolerant persisters vs. Non-persisters
Count
p adjust
Suppressed
Sphingolipid metabolism
Complement cascade
Gene ratio
B
MDA-MB-231
CDK4/6
CDK2
G1/S
S/G2
Mitosis
CDK activity
Cdt1 degron (norm.)
Time (h)
Time since CDk4/6i treatment (h)
C
MDA-MB-231
MCF-7
CDK4/6 activity
CDK2 activity
Cdt1 degron
DMSO
CDK4/6i
CDK7i (SY5609)
CDK7i (LDC4297)
CDK4/6i +CDK7i (SY5609)
CDK4/6i +CDK7i (LDC4297)
Palbo
SY
LDC
Palbo+SY
Palbo+LDC
Number of cell
Time since treatment (h)
Proliferating (89%)
Proliferating (96%)
Persisters (48%)
Persisters (45%)
Persisters (62%)
Persisters (73%)
Persisters (72%)
Persisters (81%)
Persisters (3%)
Persisters (7%)
Persisters (0.4%)
Persisters (1%)

Supplemental Figure 4. Combined CDK4/6 and CDK7 inhibition suppresses the emergence of drug-tolerant persister cells

(**A**) Dot plot showing significantly downregulated Reactome gene sets in persister versus non-persister cells following 14-day palbociclib (1 μM) treatment. Significant pathways were defined by adjusted $p < 0.05$ and FDR < 0.25. Dot color and size represent adjusted *p*-values and gene count, respectively.

(**B**) Representative single-cell traces for CDK4/6 and CDK2 activity and APC/C-degron intensity in MDA-MB-231 cells treated with or without palbociclib (1 μM). Traces were selected from one representative independent experiment ($n = 3$ biological replicates).

(**C**) Heatmaps of single-cell traces for CDK4/6 and CDK2 activity and Cdt1-degron intensity in MDA-MB-231 (left) and MCF-7 (right) cells treated with DMSO, palbociclib (1 μM), SY5609 (50 nM), LDC4297 (50 nM), palbociclib+SY5609, and palbociclib+LDC4297. Percentages indicate the proportion of persister cells (CDK2 activity > 1.0 for over 4 h during 30–48 h post-treatment). Arrows and black dotted lines indicate the start of drug treatment.

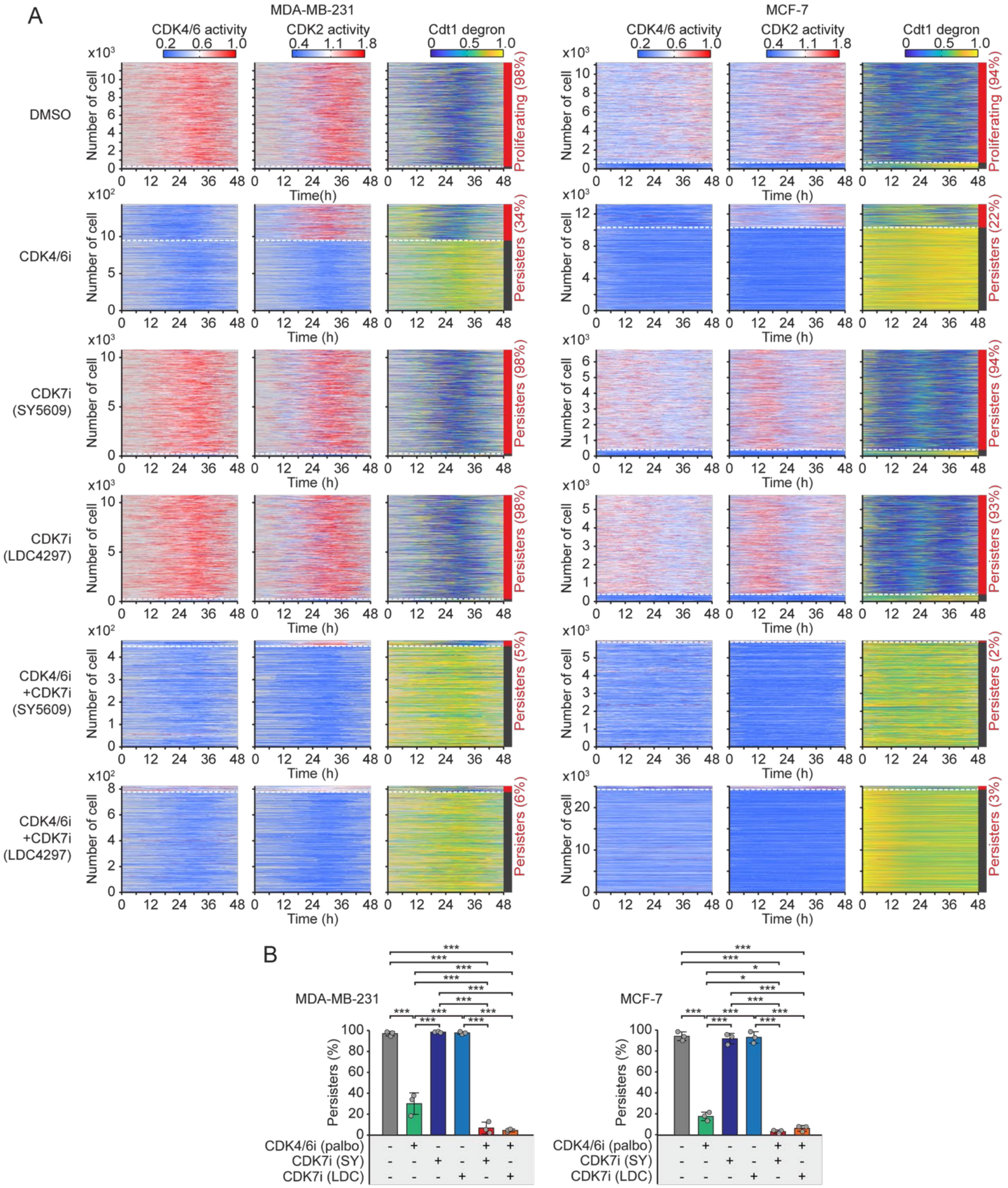

Supplemental Figure 5. Combined CDK4/6 and CDK7 inhibition suppresses the development of persister cells following prolonged treatment

(**A**) Heatmaps of single-cell traces for CDK4/6 and CDK2 activity and Cdt1-degron intensity in MDA-MB-231 (left) and MCF-7 (right) cells treated for 14 days with DMSO, palbociclib (1 μM), SY5609 (50 nM), LDC4297 (50 nM), palbociclib+SY5609, and palbociclib+LDC4297.

Percentages indicate the fraction of persister cells (CDK2 activity > 1.0 for over 4 h during 30–48 h post-treatment).

(**B**) Quantification of persister cell percentages across treatments after 14-day treatments in MDA-MB-231 and MCF-7 cells. Data are shown as means ± SD (n = 3 biological replicates). P values were calculated by one-way ANOVA with post hoc Tukey's test (* $p \leq 0.05$; *** $p \leq 0.0001$).

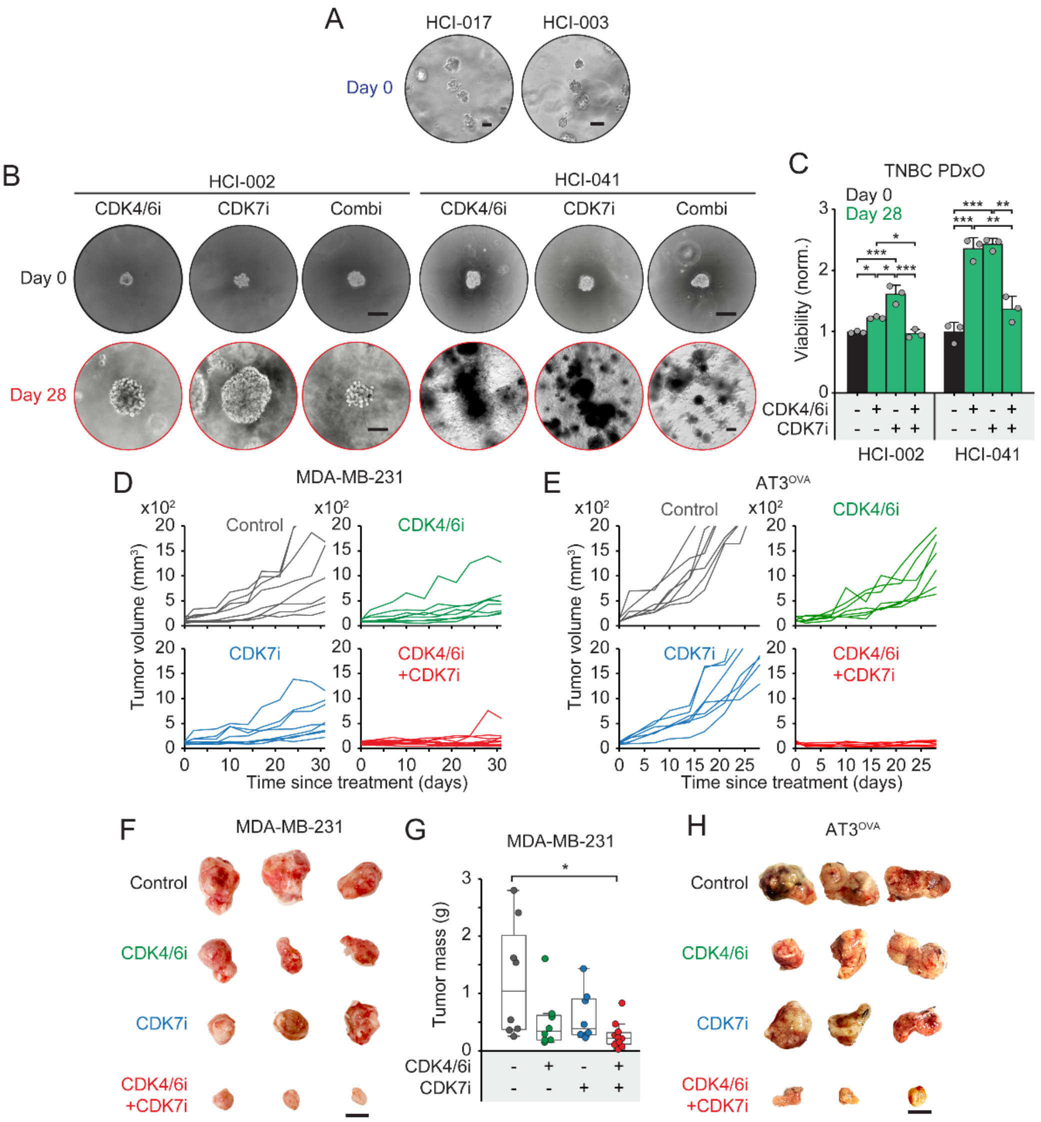

Supplemental Figure 6. Combined CDK4/6 and CDK7 inhibition effectively suppresses breast cancer growth

(**A**) Representative brightfield images of $HR^+/HER2^-$ PDxOs (HCI-017 and HCI-003) at day 0. Images were selected from one representative independent experiment ($n = 3$ biological replicates). Scale bar is 200 μm.

(**B**) Representative brightfield images of TNBC PDxOs (HCI-002 and HCI-041) treated with palbociclib (1 μM), SY5609 (50 nM), or their combination at day 0 and 28. Images were selected from one representative independent experiment ($n = 3$ biological replicates). Scale bar is 200 μm.

(**C**) Quantification of PDxO viability at day 0 and 28. Data are shown as means ± SD ($n = 3$ biological replicates). P values were calculated by one-way ANOVA with post hoc Tukey's test (* $p \leq 0.05$; ** $p \leq 0.001$; *** $p \leq 0.0001$).

(**D** and **E**) Tumor growth curves for MDA-MB-231 xenograft (**D**) and $AT3^{OVA}$ syngeneic (**E**) models treated with vehicle, palbociclib (50 mg/kg), SY5609 (2 mg/kg), or the combination.

(**F**) Representative images of harvested MDA-MB-231 tumors from indicated regimens ($n = 12$ mice for CDK4/6i+CDK7i, 8 mice for other groups). Scale bar is 1 cm.

(**G**) Boxplot showing final tumor mass for MDA-MB-231 xenografts across treatment groups. The middle line indicates the median, with box edges representing interquartile ranges (n = 12 mice for CDK4/6i+CDK7i, 8 mice for other groups). P values were calculated by one-way ANOVA with post hoc Tukey's test (* $p \leq 0.05$).
(**H**) Representative images of harvested AT3^{OVA} tumors from indicated regimens (n = 7 mice/group). Scale bar is 1 cm.

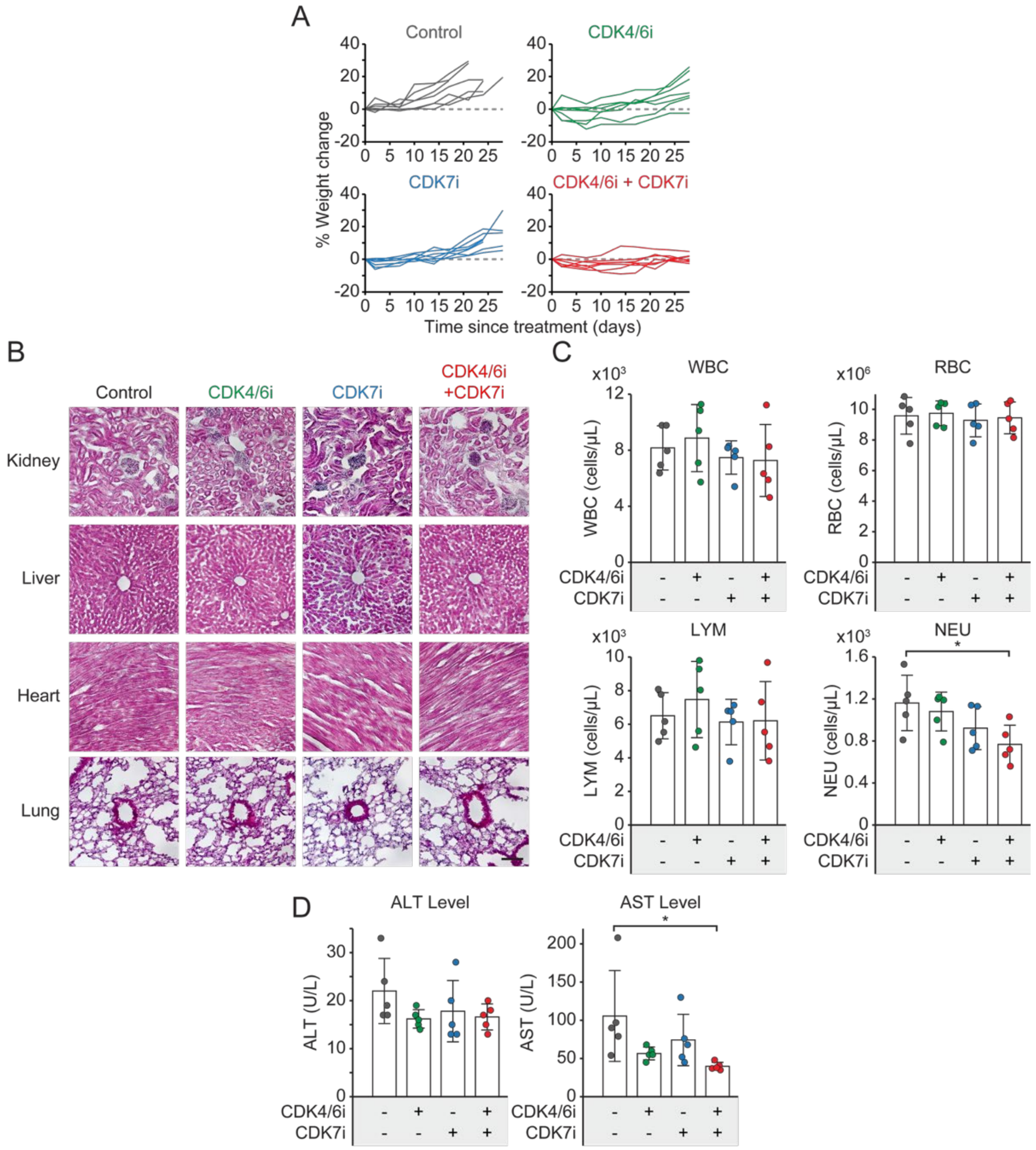

Supplemental Figure 7. Evaluation of systemic toxicity following CDK4/6i and CDK7i combination therapy

(**A**) Percentage weight change for AT3^{OVA} syngeneic models treated with vehicle, palbociclib (50 mg/kg), SY5609 (2 mg/kg), or the combination. Body weight was monitored throughout the 28-day treatment period.

(**B**) Representative H&E staining of kidney, liver, heart, and lung sections from one mouse in each treatment group, following 28 days of the indicated therapies (n = 5 mice/group). Scale bar is 100 µm.

(**C**) Quantification of hematologic parameters, including white blood cells (WBC), red blood cells (RBC), lymphocytes (LYM), and neutrophils (NEU).

(**D**) Quantification of plasma levels of alanine aminotransferase (ALT) and aspartate aminotransferase (AST) was performed to assess potential liver toxicity.

(**C** and **D**) Peripheral blood was collected after 28 days of drug treatment. Data are shown as means ± SD (*n* = 5 mice/group). *P* values were calculated by one-way ANOVA with multiple comparison Dunnett's test (* $p \leq 0.05$).

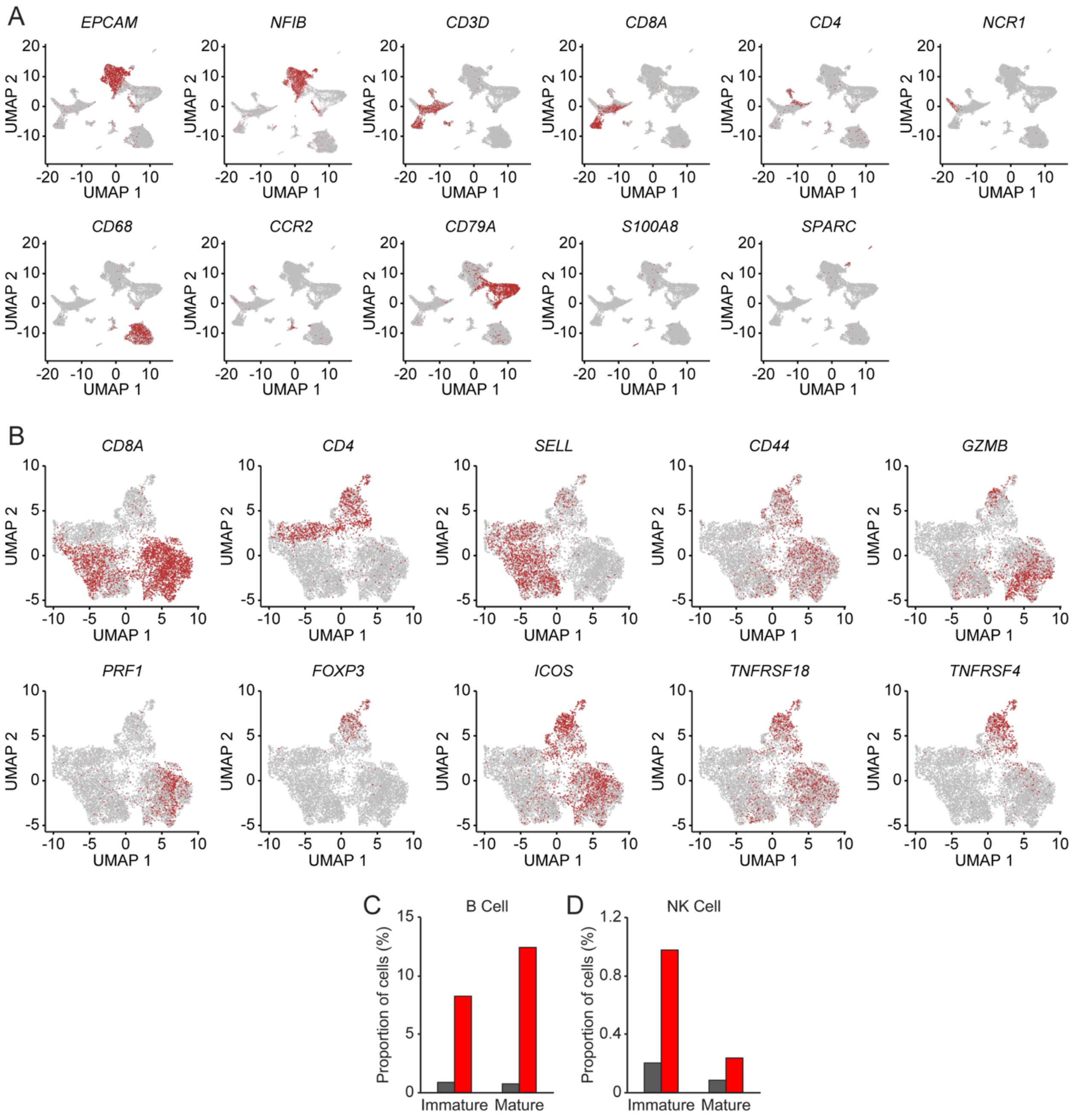

Supplemental Figure 8. Canonical marker expression supporting cell type classification in Figure 7

(**A** and **B**) UMAP plots showing the expression of canonical markers used to define cell populations. Red dots indicate cells expressing the specified genes.

(**C** and **D**) Quantification of immature (*CD24A*$^+$ and *IGHM*$^+$) and mature (*MS4A1*$^+$, *IGHM*$^+$, and *IGHD*$^+$) B cells (**C**) or immature (*CD27*$^+$ and *CD11B*$^+$) and mature (*CD27*$^-$ and *CD11B*$^+$) NK (**D**) cells in control and combination groups.

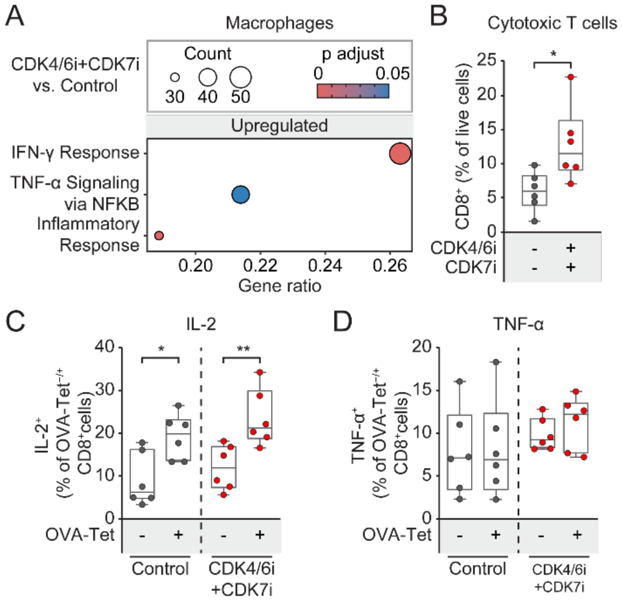

Supplemental Figure 9. Co-targeting CDK4/6 and CDK7 reprograms macrophage signaling and enhances functional activation of tumor-specific CD8$^+$ T cells

(**A**) Dot plot showing enrichment of hallmark gene sets in macrophages treated with in vivo with the combination of palbociclib (50 mg/kg) and SY5609 (2 mg/kg) compared to control. Pathways were considered significant at adjusted $p < 0.05$ and FDR < 0.25. Dot size and color reflect gene count and adjusted p values, respectively.

(**B**) Flow cytometric quantification of total CD8$^+$ T cells in AT3^{OVA} tumors following treatment.

(**C** and **D**) Flow cytometric quantification of IL-2$^+$ (**C**) and TNF-α$^+$ (**D**) producing CD8$^+$ T cells in AT3^{OVA} tumors, stratified by OVA-tetramer expression.

(**B**–**D**) The middle line indicates the median, with box edges representing interquartile ranges (n = 6 mice/group). P values were calculated by unpaired t-test (* $p \leq 0.05$; ** $p \leq 0.001$).

Supplemental Table 1. **The list of antibodies used in Fig 6A−D, 8C−D, S8A−C, and S8B−D.**

Antibody	Clone	Dilution	Source	Catalog
TCR-β – Brilliant Violet 421	H57-597	1:100	BioLegend	109229
CD4 – Brilliant Violet 605	GK1.5	1:400	BioLegend	100451
CD8a – Brilliant Violet 650	53-6.7	1:400	BioLegend	100741
NK-1.1 – Brilliant Violet 785	PK136	1:100	BioLegend	108749
F4/80 – KIRAVIA Blue 520	BM8	1:50	BioLegend	123162
CD45 – PerCP/Cyanine5.5	30-F11	1:400	BioLegend	103131
Ly-6C – PE	HK1.4	1:400	BioLegend	128007
CD11b – Alexa Fluor 700	M1/70	1:200	BioLegend	101222
Ly-6G – APC/Fire 750	1A8	1:400	BioLegend	127651
CD45 – FITC	30-F11	1:200	BioLegend	103107
FOXP3 – Alexa Fluor 700	MF-14	1:100	BioLegend	126421
TNF-α – Brilliant Violet 650	MP6-XT22	1:40	BioLegend	506333
IL-2 – PerCP-Cyanine5.5	JES6-5H4	1:20	BioLegend	503822
IFN-γ – PE/Cyanine7	XMG1.2	1:20	BioLegend	505826
OVA H-2K^b – PE	25-D1.16	1:20	MBL Life Science	TS-5001-1C
CD8a – FITC	KT15	1:20	Invitrogen	MA5-16759
CD45 – Brilliant Ultraviolet 395	30-F11	1:100	BD Bioscience	564279
