# Supplementary material for: Dual targeting of CDK4/6 and CDK7 augments tumor response and antitumor immunity in breast cancer models
Source: J Clin Invest. 2025 Aug 12;135(20):e188839. doi: 10.1172/JCI188839 (PMC12520673; doi:10.1172/JCI188839)

Full unedited blot for

Supplement Figure 2A, B, and C (MDA-MB-231 and MCF-7)

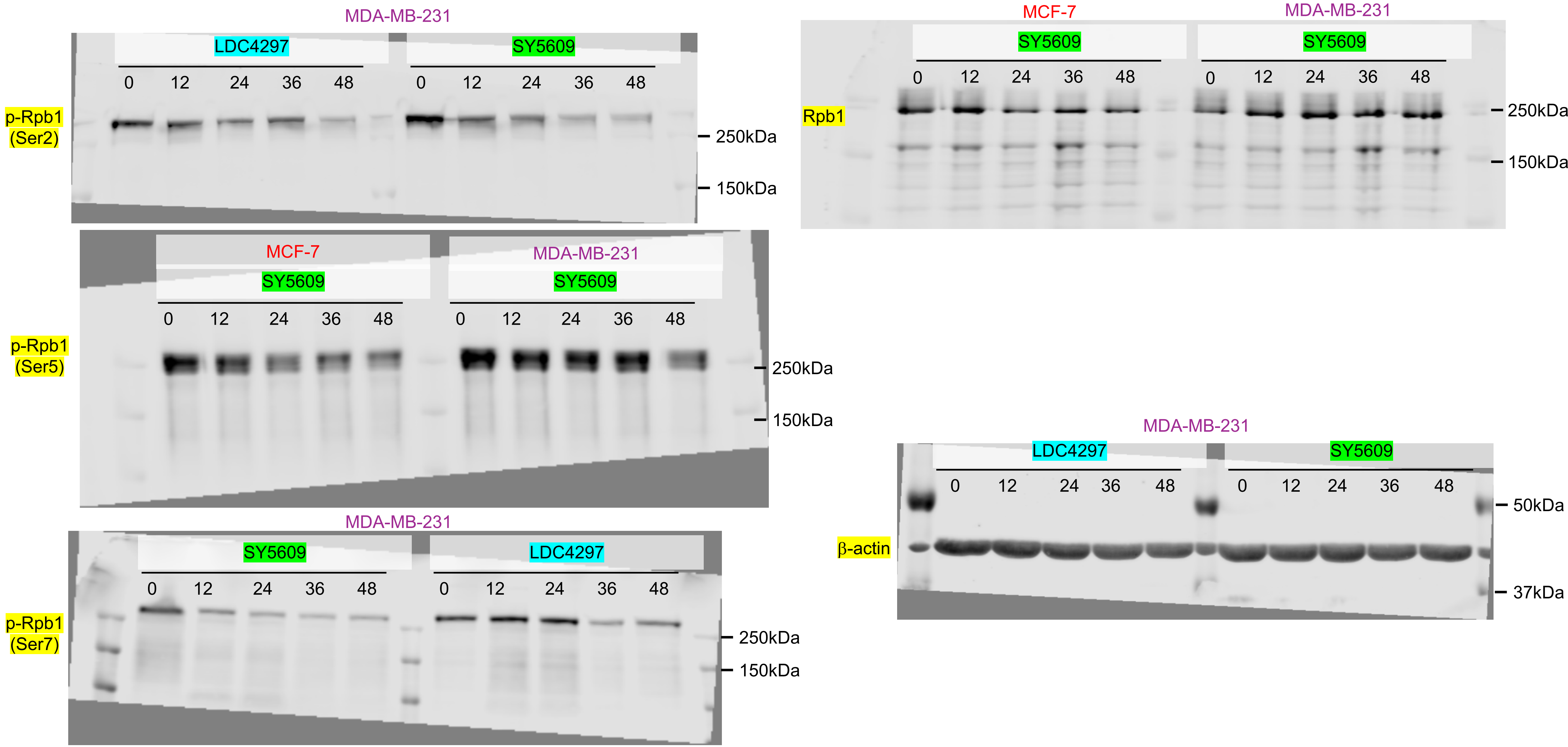

Full unedited blot for

Supplement Figure 2A, B, and C (MDA-MB-231 and MCF-7)

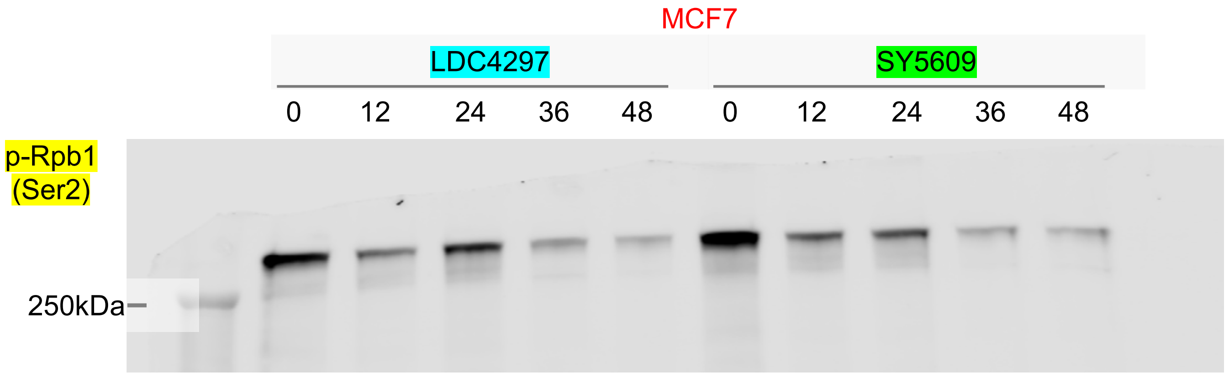

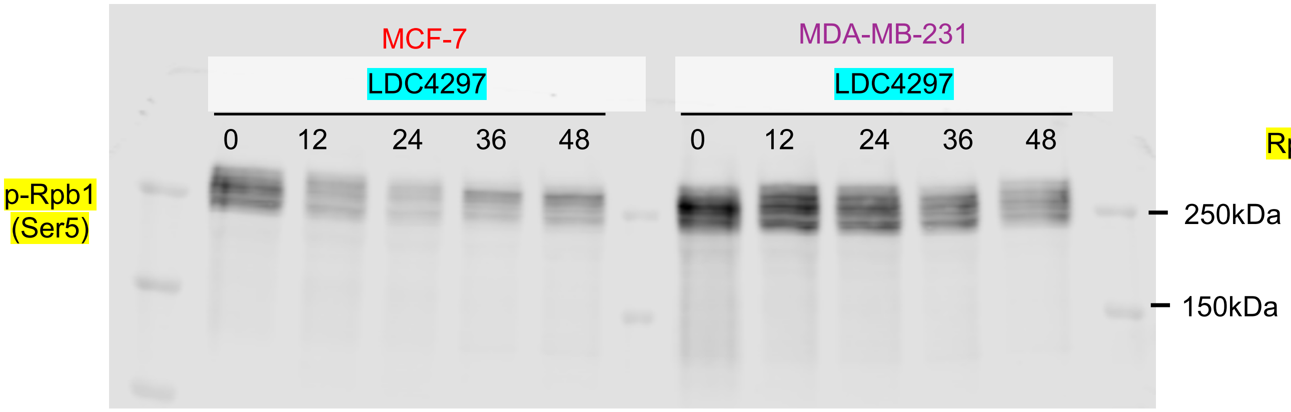

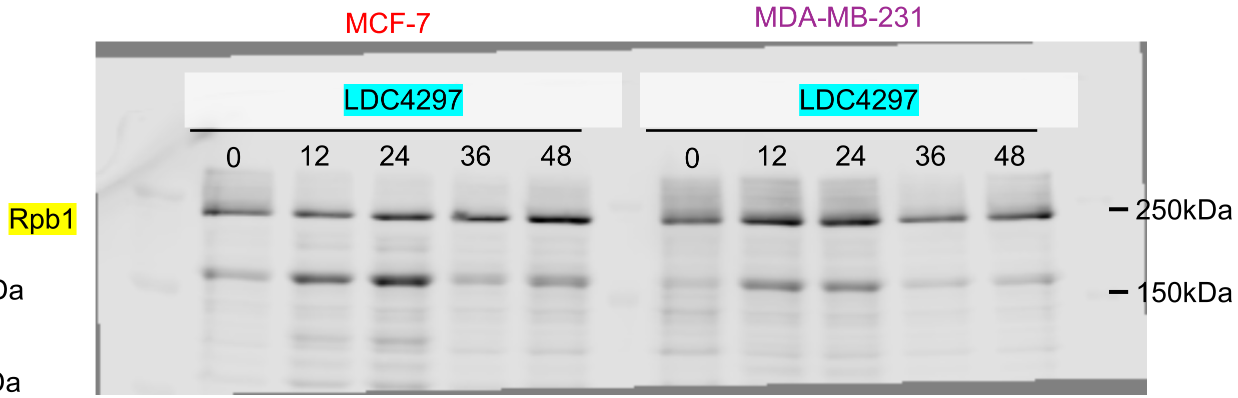

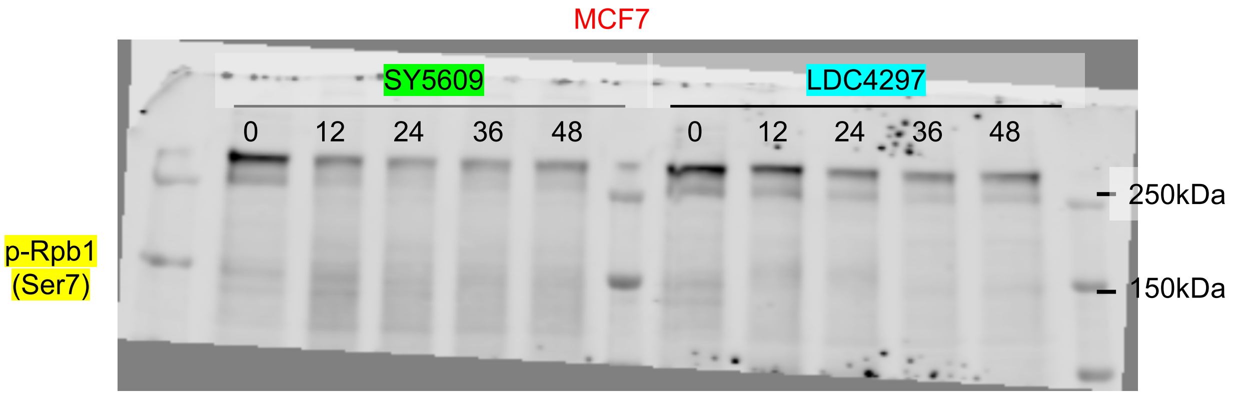

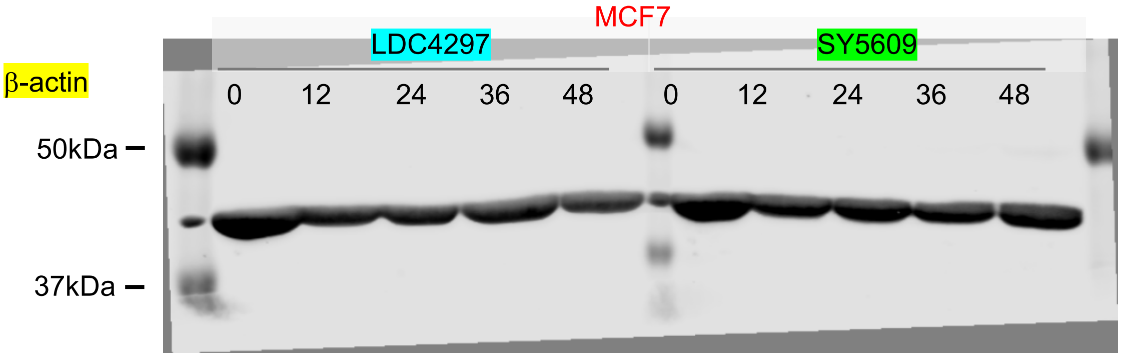

Full unedited blot for

Supplement Figure 2A, B, and C (CAMA-1)

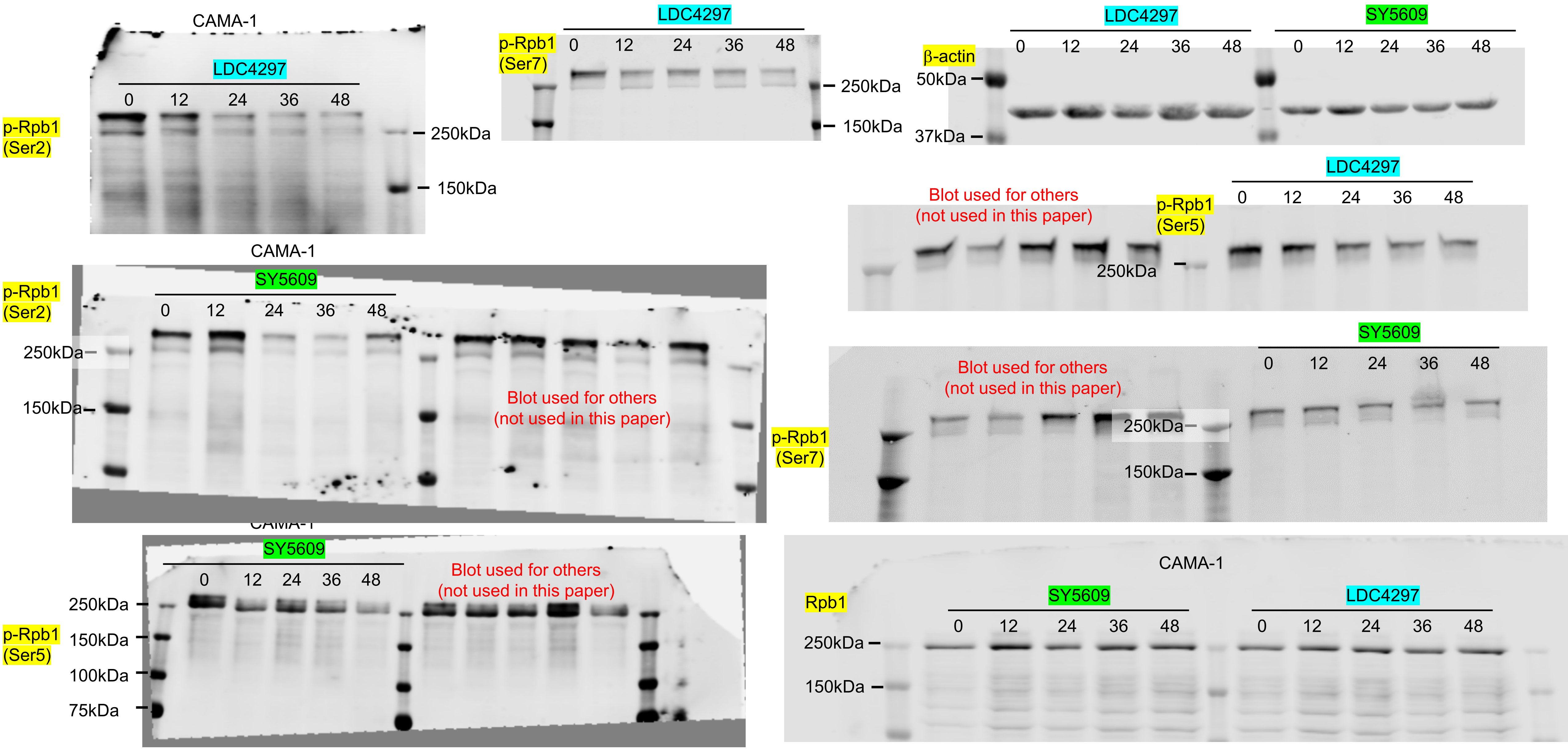

Full unedited blot for **Figure 2E**

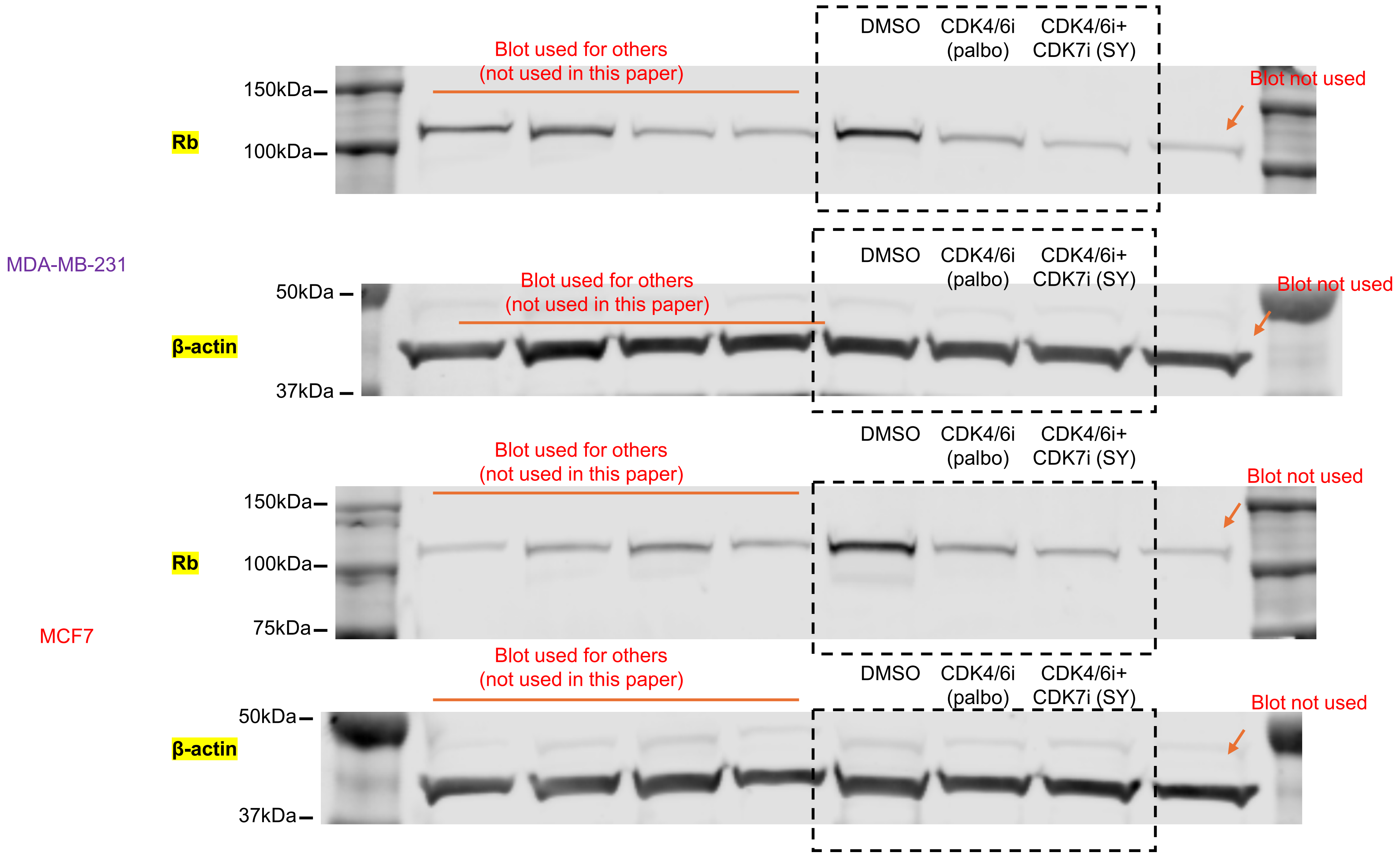

Supplement: Unedited blot and gel images [file jci-135-188839-s114.pdf]
